# Spatial Patterns of the Marine Alien Gastropod *Rapana venosa* Invasion Across the Black Sea, Mediterranean, and Atlantic Europe

**DOI:** 10.3390/biology15131012

**Published:** 2026-06-25

**Authors:** Luca Castriota, Patrizia Perzia

**Affiliations:** Unit for Conservation Management and Sustainable Use of Fish and Marine Resources, Department for the Monitoring and Protection of the Environment and for the Conservation of Biodiversity, Italian Institute for Environmental Protection and Research, Lungomare Cristoforo Colombo 4521 (Ex Complesso Roosevelt), Località Addaura, 90149 Palermo, Italy; luca.castriota@isprambiente.it

**Keywords:** alien rapa whelk, hotspots, invasive alien species, marine bioinvasion, spatiotemporal statistics, spread dynamics

## Abstract

The invasion of the marine alien gastropod *Rapana venosa* (Valenciennes, 1846) across different basins is investigated through a spatiotemporal analysis of distribution patterns, aggregation processes, and spatial structure. Occurrence data from scientific literature and citizen science were integrated with GIS-based spatial statistics to compare invasion dynamics in the Black Sea, the Mediterranean Sea, and Northwest Europe. The Black Sea represents the most advanced invasion stage, characterized by extensive distribution, multiple aggregation zones, and strong associations with brackish, nutrient-rich areas influenced by major river outflows. In the Mediterranean, the invasion has progressed from a prolonged establishment phase to a recent acceleration, with the Adriatic Sea acting as the historical core of expansion. Here, persistent populations are concentrated near the Po River delta and lagoon systems, where reduced salinity and high nutrient loads favor both settlement and long-term persistence. In Northwest Europe, *R. venosa* remains in the establishment phase, forming a compact and localized nucleus along the French Atlantic coast without evidence of broad spatial expansion. Our analyses suggest that environmental factors, particularly salinity gradients and riverine inputs, are possibly related to the observed invasion patterns. Transitional coastal environments emerge as important areas for establishment and subsequent spread, suggesting that monitoring efforts should prioritize these environments.

## Simple Summary

*Rapana venosa* is an invasive marine snail that has spread across several marine regions of the world. In this study, we analyzed where and how this species has expanded in western Eurasia (up to the Black Sea) by combining published records and citizen science data with spatial analysis tools. We found that the invasion is most advanced in the Black Sea, where the species is widespread and linked to brackish, nutrient-rich areas influenced by rivers. In the Mediterranean Sea, the invasion has only recently accelerated, with the north-western Adriatic Sea representing the main area of expansion. In Northwest Europe, the species is still limited to a small area and has not yet spread widely. Overall, our results suggest that environmental conditions, especially salinity and river inputs, play a key role in determining where the species can establish and spread. Coastal areas influenced by freshwater emerge as particularly important, highlighting the need for monitoring and management to reduce potential impacts.

## 1. Introduction

*Rapana venosa* (Valenciennes, 1846), commonly known as the veined rapa whelk, is a large predatory gastropod native to the temperate waters of the northwestern Pacific, including the Sea of Japan, Yellow Sea, and East China Sea. Since the mid-20th century, this species has expanded far beyond its native range, largely facilitated by maritime traffic—through transport via ballast water or as part of the hull fouling community—and the accidental transfer of egg masses associated with aquaculture activities [1]. The first record of *R. venosa* outside its native distribution occurred in 1947 in the Black Sea, in Novorossiysk Bay, misidentified as *R. bezoar* (Linnaeus, 1767) [2]. Within a decade, the species spread along the Caucasian and Crimean coasts and penetrated the Sea of Azov [3], demonstrating a remarkable capacity for rapid dispersal and establishment in new environments. Today, *R. venosa* is regarded as one of the most dangerous invasive species in both the Black Sea and the Mediterranean, owing to its intense predatory pressure on native bivalve populations and its ability to modify benthic community structure. At the same time, it has also become a valuable fishery resource in several regions, particularly within the Black Sea basin [4,5]. Although the species has been reported in the Sea of Marmara since 1966 [6] and later detected in several Mediterranean coastal regions, including parts of France, the mechanism underlying its expansion within the Mediterranean Sea remains unclear. It is nevertheless considered very likely that this distribution pattern results from multiple independent introduction events rather than from a single, continuous colonization process. In several parts of the Mediterranean Sea, however, *R. venosa* shows a more limited degree of invasion compared to its extensive and well-documented spread in the Black Sea. Parallel to its Mediterranean spread, *R. venosa* has also invaded the eastern Atlantic, where individuals have been recorded from the North Sea to the coasts of Spain, as well as in the western Atlantic, where it was first detected and established in Chesapeake Bay in 1998 [7]. A particularly successful and well-documented invasion took place in the Río de la Plata estuary (western Atlantic) beginning in 1999. Since then, the species has expanded along the Argentine and Uruguayan coasts and, more recently, into southern Brazil, where it impacts native mollusk communities and exhibits high ecological adaptability [8,9]. Globally, the invasive success of *R. venosa* is attributed to its ecological plasticity, broad dietary spectrum, high fecundity, long lifespan, strong tolerance to human-induced environmental stressors, and ability to exploit a wide range of habitats—from soft substrates to mussel and oyster beds—consistently generating significant impacts on native benthic communities and commercial fisheries [1]. As such, *R. venosa* represents an important model organism for studying marine biological invasions and for developing effective management strategies across invaded areas. Moreover, understanding the spread and ecological impact of *R. venosa* greatly benefits from the application of spatial statistics and GIS-based analyses, which enable the identification of invasion hotspots, the modeling of dispersal pathways, and the assessment of environmental drivers shaping its distribution [10,11,12,13,14,15]. In this paper, we present an analysis of the spatial distribution of *R. venosa* across the Black Sea and adjacent waters, the Mediterranean Sea, and the northwest Atlantic, with the objective of identifying the directional patterns that characterize its spread and assessing the geographic factors that may have influenced its invasion dynamics.

## 2. Materials and Methods

### 2.1. Rapana venosa Invasion History

To investigate the distribution of *Rapana venosa* in the Black Sea and adjacent waters (the Azov and Marmara Seas) (BS), the Mediterranean Sea (MS), and Northwest European waters (NWE), occurrence records were compiled from both the scientific literature and citizen-science archives. Relevant studies were identified using the search engines Google (google.com), Google Scholar (scholar.google.com), and ResearchGate (researchgate.net), with searches conducted up to December 2025. A range of keyword combinations was employed to ensure comprehensive coverage of the available literature. Terms such as *Rapana venosa*, its synonym *Rapana thomasiana*, the misapplied name *Rapana bezoar*, and its common names “rapa whelk” and “veined rapa whelk” were used as search keywords, individually and in combination with geographical descriptors including Black Sea, Azov, Marmara, Mediterranean, and Atlantic. Additional bibliographic sources were gathered by examining the reference lists of the retrieved publications. Citizen-science archives (i.e., iNaturalist) were also consulted.

The integration of occurrence data from heterogeneous sources, including citizen-science platforms and literature, may introduce spatial and temporal biases associated with uneven sampling effort, reporting intensity, and temporal coverage. Such heterogeneity can potentially affect the robustness and interpretation of inferred invasion patterns. To reduce these limitations, a filtering protocol was applied: (i) only records with precise geographic coordinates or clearly identifiable sampling locations were retained; (ii) duplicate, doubtful, or taxonomically uncertain records were excluded; and (iii) for citizen-science data, only records accompanied by clear photographs were considered, and exclusively those documenting live individuals or specimens retaining the operculum were included.

An unpublished observation by one of the authors (LC), at coordinates 44.845398° N, 12.435761° E, was also considered.

The compiled dataset included the year of the record (or, when unavailable, the publication year), locality, country, geographic coordinates, and source.

### 2.2. Distribution, Aggregation Patterns, and Spatial Structure Analyses

Spatial data were processed in ArcMap 10.3 (ESRI, Redlands, CA, USA) by retaining only the earliest occurrence record within each 0.05° latitude–longitude grid cell, following the approach of Lipej et al. [16]. This procedure ensured that only a single occurrence was assigned to intensively sampled areas, thereby reducing the effects of preferential sampling and the potential bias associated with distribution analyses [16]. The resulting subset was used for all analyses, regardless of the number of individuals recorded within each grid cell.

Following Perzia et al. [11], spatial and temporal statistical analyses were performed to characterize the distribution, aggregation patterns, and spatial structure of the records to identify (i) spatial distribution patterns, (ii) temporal changes in distribution, (iii) the earliest and most recent phases of species spread, (iv) dispersal versus settlement areas, and (v) spatial outliers. Qualitative and quantitative multi-parameter modeling was carried out using the Spatial Analyst and Spatial Statistics toolboxes in ArcMap.

Data were analyzed separately for the three investigated areas—BS, MS, and NWE—due to temporal and geographical differences in the invasion process.

Invasion phases of *R. venosa* were identified from the cumulative occurrence curve, segmented by changes in slope, following Perzia et al. [11]. Regression lines were used to estimate the rate of increase and to delineate the different phases of the invasion process, i.e., arrival (Ar), establishment (Es), and expansion (Ex). The *y*-axis values also represent the expansion area, measured as the cumulative number of 0.05° latitude/longitude grid cells affected.

Spatial patterns in *R. venosa* occurrences were investigated using a combination of complementary spatial statistical approaches.

Cumulative kernel density analyses were applied to qualitatively explore temporal and spatial variations in occurrence density across different time periods [11,12,13,14,15,17]. Specifically, the analyses were conducted to (i) evaluate temporal and spatial increases in occurrence density, (ii) identify areas of species expansion, and (iii) detect persistently high occurrence densities.

Key distribution characteristics—center of gravity (median center), directional dispersion along the east–west axis (XStdDist) and along the north–south axis (YStdDist), and directional trends [18,19]—were calculated for each invasion phase within the study areas.

The north–south and east–west components reported in this study do not represent actual dispersal pathways but are used solely as statistical descriptors of the spatial configuration of the distribution. This approach enabled the assessment and comparison of spatiotemporal distribution changes and the evaluation of shifts in distribution shape (e.g., dispersed, compact, or elongated), thereby helping to identify patterns of species expansion and/or contraction over time [11,12,13,14,15].

Incremental Spatial Autocorrelation (ISA) was first conducted to determine the distance at which spatial autocorrelation in occurrence density was maximized, based on peaks in the z-score of Global Moran’s I [20]. The ISA analysis also identified the optimal distance threshold used to define the spatial scale of subsequent local analyses.

Hot spot analysis was performed using the Getis–Ord Gi* statistic (GOG*) to identify statistically significant spatial clusters of high (hot spots) and low (cold spots) occurrence densities by comparing local neighborhood values with the global spatial context [21]. Years of occurrence were used as the feature attribute, with clusters classified according to their confidence level: high-confidence hot spots indicate recent occurrence clusters, high-confidence cold spots indicate older clusters, and clusters not statistically significant indicate random spatial patterns. This approach was applied to assess spatial trends in species spread and settlement dynamics [11,12,13,14,15].

Finally, local spatial autocorrelation was assessed using Anselin’s Local Moran’s I (AMI) to identify statistically significant spatial outliers in species occurrence [22]. In particular, high–low and low–high outliers were detected, representing recent records located near clusters of older records, and vice versa, reflecting negative spatial autocorrelation [11,12,13,14,15].

Table 1 summarizes the analyses and indicators applied to the study areas (BS, MS, and NWE).

## 3. Results

A total of 810 occurrence records of *Rapana venosa* were compiled from the Black Sea and adjacent waters (562), the Mediterranean Sea (176), and Northwest Europe (72). The full dataset is provided as Supplementary Material Table S1 [1,2,3,4,5,6,23,24,25,26,27,28,29,30,31,32,33,34,35,36,37,38,39,40,41,42,43,44,45,46,47,48,49,50,51,52,53,54,55,56,57,58,59,60,61,62,63,64,65,66,67,68,69,70,71,72,73,74,75,76,77,78,79,80,81,82,83,84,85,86,87,88,89,90,91,92,93,94,95,96,97,98,99,100,101,102,103,104,105,106,107,108,109,110,111,112,113,114,115,116,117,118,119,120,121,122,123,124,125,126,127,128,129,130,131,132,133,134,135,136,137,138,139,140,141,142,143,144,145,146,147,148,149,150,151,152,153,154,155,156,157,158,159,160,161,162,163,164,165,166,167,168,169,170,171,172,173,174,175,176,177,178,179,180,181,182,183,184,185,186,187,188,189,190,191,192,193,194,195,196,197,198,199,200,201,202,203].

After filtering, 453 records were retained for analysis, including 319 from BS, 95 from MS, and 39 from NWE.

**Figure 1:**
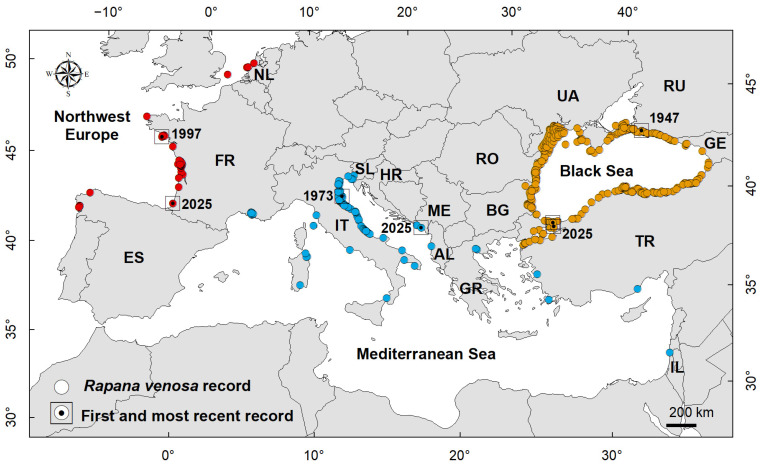
Distribution of *Rapana venosa* across the three invasion areas: the Black Sea and its adjacent waters (Azov and Marmara Seas) (orange dots), the Mediterranean Sea (blue dots), and Northwest Europe (red dots). The first and most recent records in each area are also reported.

Figure 1 shows the spatial distribution of records, as well as the first and most recent occurrences in each area: the Russian coast (1947) and the Bosphorus Strait (2025) in the BS area; the Adriatic Sea (1973) and Montenegro (2025) in the MS; and the French Atlantic coast (1997 and 2025) in the NWE.

### 3.1. Invasion History and Spatial–Temporal Patterns of Rapana venosa Distribution

**Figure 2:**
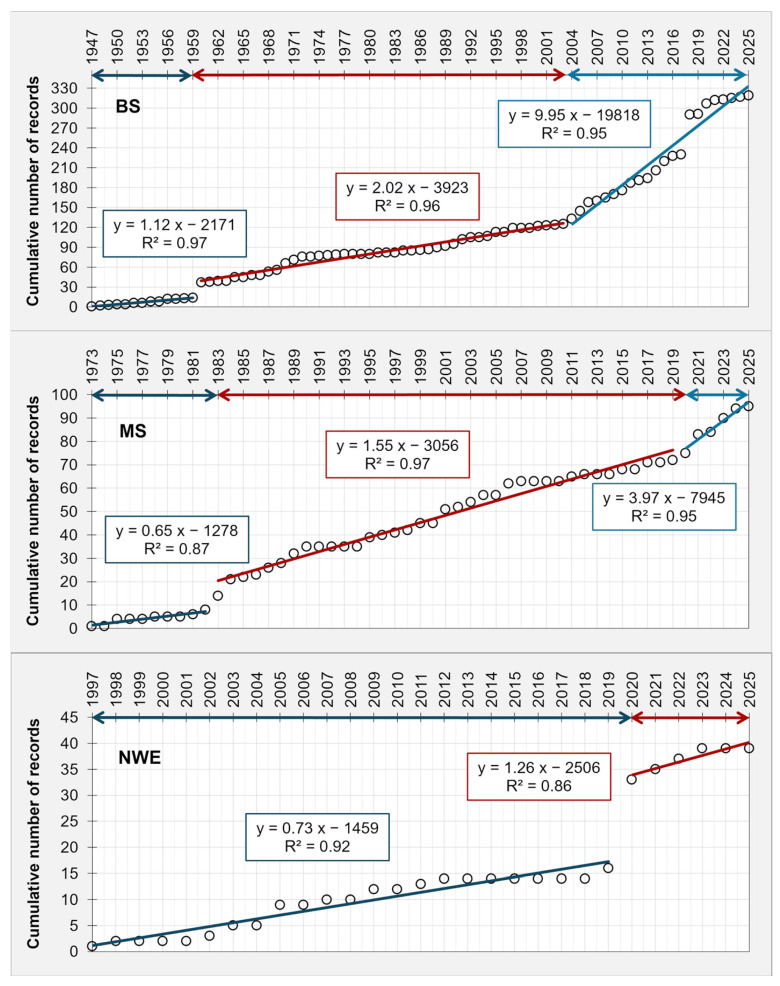
Cumulative curves of occurrences of *Rapana venosa* in the three invasion areas: the Black Sea and adjacent waters (the Azov and Marmara seas) (BS), the Mediterranean Sea (MS), and Northwest Europe (NWE). Only the first records within a 0.05° Lat/Long grid were considered. Arrows indicate the phases in the invasion process—arrival (blue), establishment (red), and expansion (light blue)—and mark the corresponding years for each phase.

Figure 2 shows the cumulative occurrence curves of *R. venosa* in the three invasion areas (BS, MS, and NWE), together with the cumulative number of grid cells affected by the species over time. The equations of the regression lines and the corresponding R^2^ values are also reported.

In the BS area, three slope changes were identified, corresponding to the arrival phase (1947–1959), the establishment phase (1960–2003), and the expansion phase (2004–2025). The marked increase in slope values indicates that the invasion of *R. venosa* in the BS area has accelerated substantially during the last 21 years, with a slope of 9.95 ± 0.51 during the expansion phase, compared to much lower rates during the arrival (slope = 1.12 ± 0.06) and establishment phases (slope = 2.02 ± 0.06). The progressive increase in slope values indicates an acceleration of the invasion process over time, with an intensification during the most recent expansion phase.

Similarly, in the MS area, the cumulative curve displayed three clear slope changes, corresponding to: (i) the arrival phase (1973–1982; slope = 0.65 ± 0.09); (ii) the establishment phase (1983–2019; slope = 1.55 ± 0.04); and (iii) the expansion phase (2020–2025; slope = 3.97 ± 0.46). In both the BS and MS areas, the arrival phase was relatively short (12 and 9 years, respectively), followed by a long-term establishment phase, lasting 43 years in the BS and 36 years in the MS.

In the NWE area, the cumulative curve showed two distinct changes in slope, corresponding to a prolonged arrival phase (1997–2019; slope = 0.73 ± 0.05) and a subsequent establishment phase (2020–2025; slope = 1.26 ± 0.25). No expansion phase was detected in this area.

**Figure 3:**
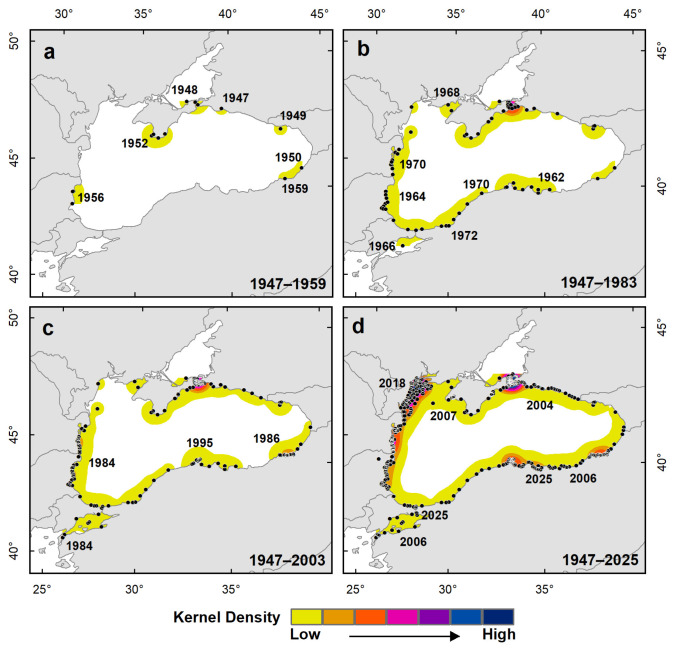
Cumulative kernel density maps showing the spatiotemporal expansion of *Rapana venosa* in the Black Sea and adjacent waters: (**a**) 1947–1959; (**b**) 1947–1983; (**c**) 1947–2003; (**d**) 1947–2025. Black circles indicate occurrence records of *R. venosa*.

**Figure 4:**
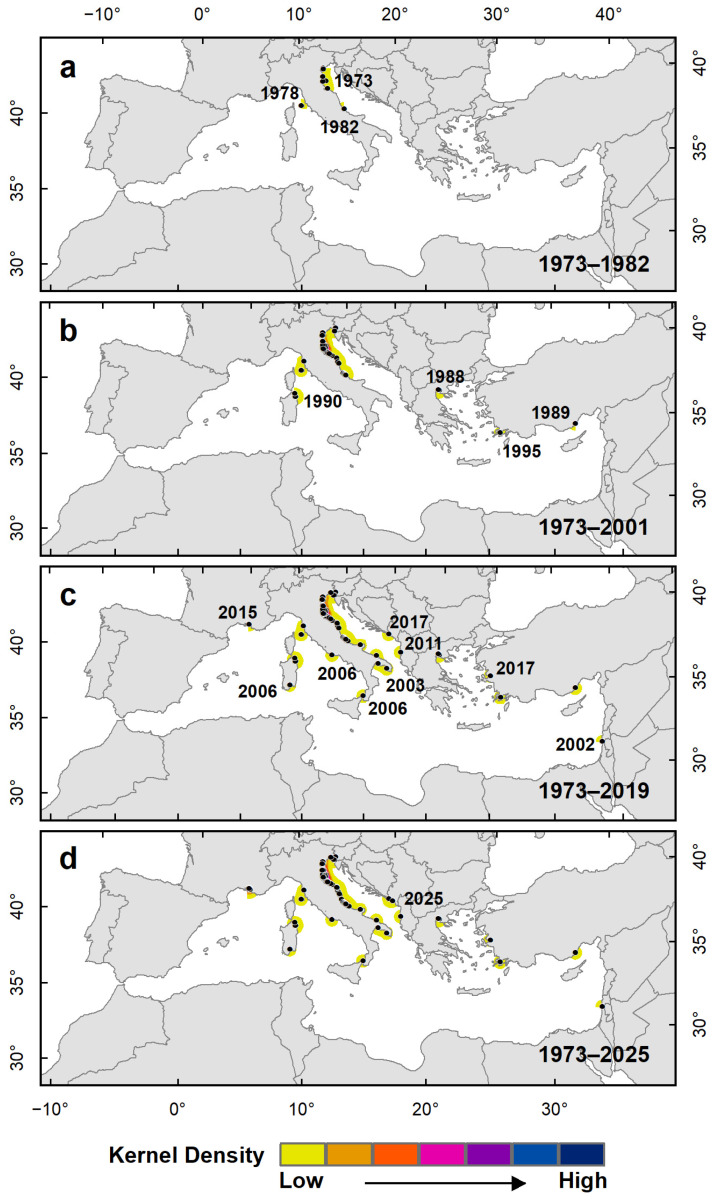
Cumulative kernel density maps showing the spatiotemporal expansion of *Rapana venosa* in the Mediterranean Sea. Maps (**a**–**d**) represent the invasion phases: (**a**) 1973–1982; (**b**) 1973–2001; (**c**) 1973–2019; (**d**) 1973–2025. Black circles indicate occurrence records of *R. venosa*.

**Figure 5:**
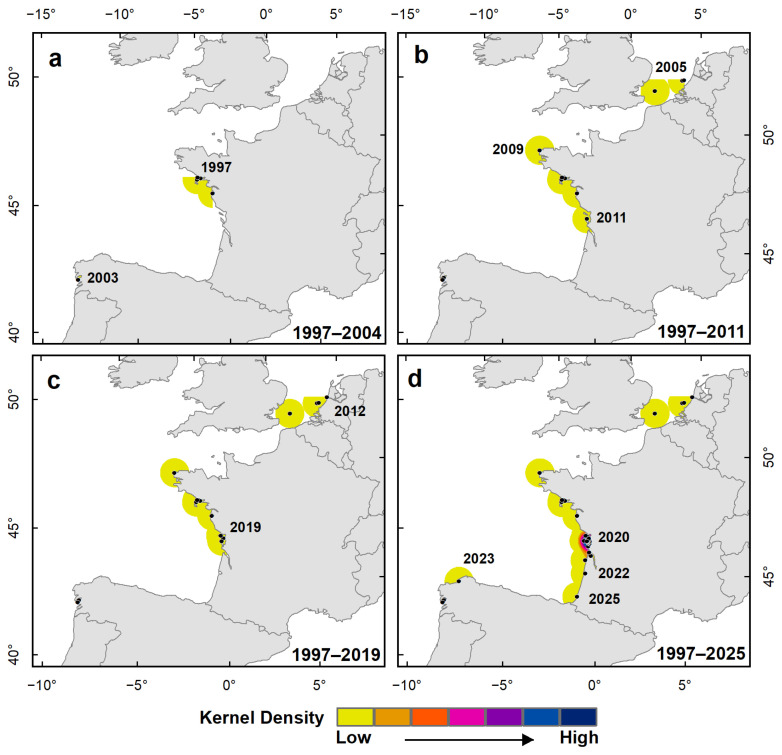
Cumulative kernel density maps showing the spatiotemporal expansion of *Rapana venosa* in northwestern Europe: (**a**) 1997–2004; (**b**) 1997–2011; (**c**) 1997–2019; (**d**) 1997–2025. Black circles indicate occurrence records of *R. venosa*.

Figure 3, Figure 4 and Figure 5 illustrate the spatiotemporal expansion of *R. venosa* in the BS, MS, and NWE areas, respectively, based on cumulative occurrence records and kernel density analysis. These showed a period-to-period variation in space and time, highlighting substantial changes in occurrences, both in terms of spread and species aggregation. The kernel density gradient (from low to high) highlights areas with increasing concentrations of records along the coastline.

#### 3.1.1. Black Sea and Adjacent Waters

During the arrival phase (1947–1959; Figure 3a), occurrences were limited and mainly concentrated in the northwestern Black Sea, particularly along the Crimean coast.

After 1960, the species entered the establishment phase (Figure 3b,c). During this period, the number of records increased, and the distribution extended along a larger portion of the northwestern and western coasts, with additional occurrences appearing along the southern coastline.

The most recent cumulative map (1947–2025; Figure 3d), which includes the expansion phase of the species, shows a basin-wide coastal distribution, with higher kernel density values along extensive portions of the western and southern Black Sea coasts and additional records along the northern and eastern sectors.

#### 3.1.2. Mediterranean Sea

During the arrival phase (1973–1982; Figure 4a), records are scarce and mainly concentrated in the northern Adriatic Sea, particularly along the Italian coast. An isolated occurrence is recorded in the northern Tyrrhenian Sea (1978). Kernel density values remain low and highly localized, reflecting the initial detection of the species with limited spatial spread.

In the subsequent period (1973–2001; Figure 4b), the number of records increases, and the distribution expands across much of the Adriatic basin, with additional occurrences reported in the central Mediterranean and in the eastern Mediterranean (the Aegean Sea and the south Turkish coast). Areas of higher kernel density begin to emerge along the Adriatic coasts, indicating increasing concentrations of records in this region.

In the following phase (1973–2019; Figure 4c), the spatial distribution becomes broader, with records reported throughout much of the central and eastern Mediterranean, including the Ionian Sea. The Adriatic Sea remains the main area with higher record density, while additional occurrences contribute to a more widespread distribution of records across the basin.

In the most recent cumulative phase (1973–2025; Figure 4d), higher kernel density values remain concentrated in the Adriatic Sea, while additional occurrences are reported in the western Mediterranean. Notably, the distribution of records remains largely confined to the northern Mediterranean coasts. Overall, the pattern reflects a progressive increase and spatial spread of occurrence records across the Mediterranean, while maintaining a clear concentration along its northern coastal areas.

#### 3.1.3. Northwest Europe

Figure 5 shows the progressive expansion of *R. venosa* in the NWE area from the initial detection site, with increasing spatial continuity over time.

The first three maps (Figure 5a–c) represent the arrival phase. During the earliest period (1997–2004), the species was recorded at a limited number of sites along the French Atlantic coast. In the following period (1997–2011), additional records appeared in the Bay of Biscay and the English Channel, indicating a gradual expansion from the initial introduction area. By 1997–2019, the distribution extended further along the French coast and the southern Dutch coast.

The last map (1997–2025), including the recent establishment phase, shows a more continuous distribution along parts of the French Atlantic coast, with recent records confirming the continued presence of the species in the region.

### 3.2. Key Characteristics of Distribution

**Figure 6:**
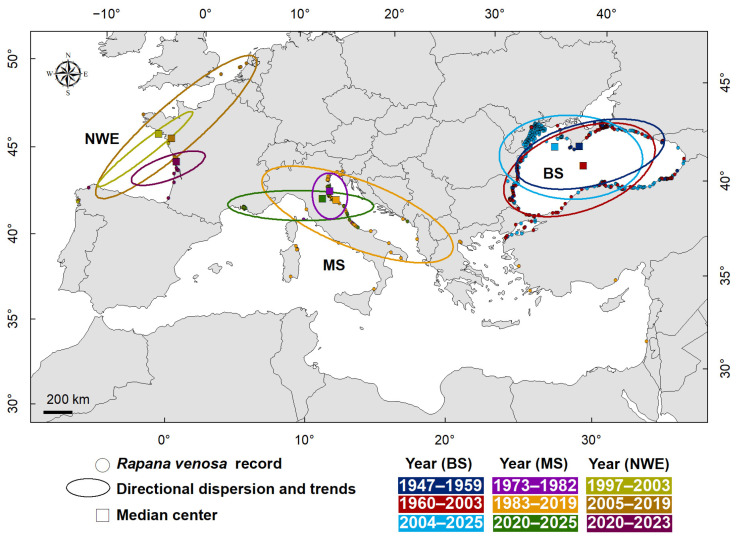
Key distribution characteristics of *Rapana venosa* in the three invasion areas: the Black Sea and adjacent waters (the Azov and Marmara seas) (BS), the Mediterranean Sea (MS), and Northwest Europe (NWE). Central tendency (median center), directional dispersion, and temporal trends calculated for different periods, showing changes in spatial distribution over time.

Figure 6 presents the key characteristics of the distribution of *R. venosa* in BS, MS, and NWE areas across the examined periods.

The central tendency, directional dispersion, and directional trend varied across space and time in the three investigated areas, revealing distinct changes in the spatial configuration of the invasion phases, particularly in MS and NWE. These patterns indicate area-specific trajectories, with periods of spread followed by partial contraction and, in some cases, a reorientation of the main dispersal axis.

#### 3.2.1. Black Sea and Adjacent Waters

In the BS, dispersion was initially higher along the north–south axis (YStdDist = 525 km) than along the east–west axis (XStdDist = 219 km) in 1947–1959 (Ar phase). During 1960–2003 (Es phase), both axes remained high and relatively stable (548 km in Y; 281 km in X), reflecting sustained broad distribution. In 2004–2025 (Ex phase), XStdDist increased substantially to 496 km, while YStdDist decreased to 290 km, indicating a shift toward greater zonal dispersion. The directional trend remained consistent across periods (70–92°), suggesting a stable main orientation of spread.

#### 3.2.2. Mediterranean Sea

In the MS, the first period (1973–1982), corresponding to the arrival phase, was characterized by moderate and relatively balanced dispersion (159 km in X; 121 km in Y), with occurrences concentrated along the northeastern coast of Italy in the Adriatic Sea. A marked spread occurred during the 1983–2019, Es phase, when XStdDist increased to 704 km and YStdDist to 231 km, indicating a pronounced zonal dispersion. In the most recent period (2020–2025, Ex phase), both axes contracted (505 km in X and 104 km in Y), suggesting a westward spatial consolidation. The rotation angle shifted from 175° in the earliest period to 111° and then 91°, indicating a progressive reorientation of the main dispersal axis. The ellipse became elongated from Italy toward France; however, the highest number of records continued to be reported in the Adriatic Sea, where the median centers of distribution remained confined.

#### 3.2.3. Northwest Europe

The arrival phase of *R. venosa* in the NWE area was examined in two sub-periods (1997–2003 and 2005–2019). Dispersion was consistently greater along the north–south axis than along the east–west axis throughout all periods. YStdDist increased from 417 km (1997–2003) to a peak of 728 km (2005–2019) before decreasing to 269 km in 2020–2025 (Es phase), indicating an initial longitudinal confinement followed by a pronounced meridional spread during the arrival phase and a subsequent contraction in the establishment phase. XStdDist followed a similar but less marked pattern (52 km → 160 km → 80 km). The directional trend remained relatively stable, with rotation values between 49° and 70°, indicating a persistent NE–SW orientation from France toward Spain despite changes in dispersion magnitude. The median centers for all periods were located along the western Atlantic coast of France.

### 3.3. Aggregation Patterns and Spatial Structure 

At the global scale, the ISA revealed statistically significant positive spatial autocorrelation in all three invasion areas, as indicated by Global Moran’s I values higher than the expected indices and z-scores exceeding 2.58 (*p* < 0.01). This confirms that occurrence records were spatially clustered rather than randomly distributed.

In the BS, the GMI (0.18) exceeded the expected value (−0.003), showing moderate spatial clustering. This intermediate pattern suggests the presence of identifiable aggregation zones combined with a relatively wide spatial spread.

In the MS, the GMI (0.11) was only slightly above the expected index (−0.011), indicating weaker but still significant clustering. This pattern reflects a broad and diffuse distribution.

In the NWE, the observed GMI (0.58) was markedly higher than the expected value (−0.026), indicating strong spatial clustering and a well-defined aggregation structure. This suggests that records were concentrated within specific sectors, consistent with a localized invasion nucleus and limited dispersal beyond core areas.

Overall, the results indicate significant but area-dependent clustering intensity, with moderate clustering in the BS, more diffuse spatial structure in the MS, and the strongest aggregation in the NWE.

**Figure 7:**
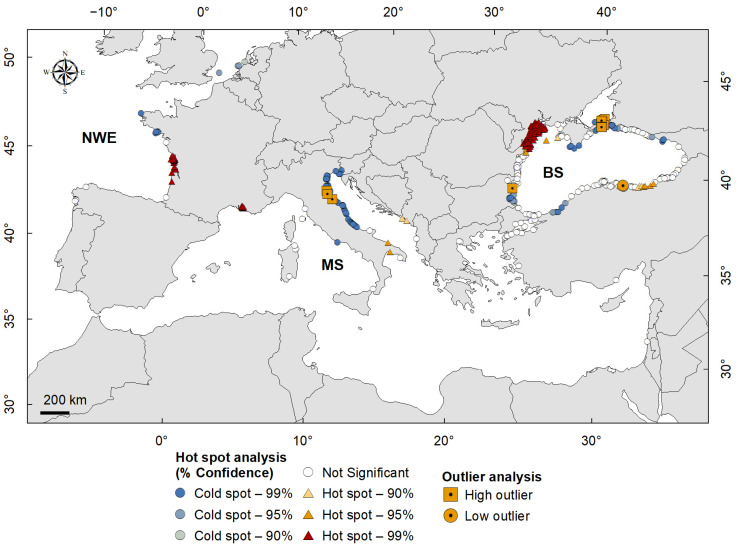
Results of the hot spot (Getis–Ord Gi*) and outlier analysis (Anselin local Moran’s I) on records of *Rapana venosa* in the three invasion areas: the Black Sea and adjacent waters (the Azov and Marmara seas) (BS), the Mediterranean Sea (MS), and Northwest Europe (NWE). Areas with statistically significant spatial clustering (cold spot—blue circles; hot spot—red circles) are shown. Low outliers (yellow circles) and high outliers (yellow squares) are indicated. White circles indicate records with non-significant index values.

At a local scale, the GOG* and ALM analyses revealed clear spatial structuring in the distribution of significant hot spots, cold spots, and outliers across the three invasion areas (Figure 7):

- Black Sea and adjacent waters—In the BS area, the earliest records are strongly clustered in the north-eastern sector (cold spot, 99%), consistent with the historical introduction area. Additional cold spots along the southern and eastern coasts denote areas of earlier establishment. Extensive and highly significant hot spots (95–99%) occur along the north-western Black Sea shelf, indicating large and persistent core zones of recent spread and ongoing expansion. The spatial arrangement of cold and hot spots supports a stepwise expansion from the north-eastern nucleus, with progressive infilling of adjacent areas and short-range dispersal processes. The presence of high outliers in the north-eastern Black Sea highlights sites with very recent records surrounded by older ones.
- Mediterranean Sea—In the MS, a significant cold spot (90–95%) is identified along the central–northern Adriatic coast, confirming this region as the primary aggregation area. Most other locations in the Mediterranean Sea were classified as not significant, indicating weak spatial clustering of similar occurrence years (i.e., a dispersed spatial pattern). A distinct pattern emerges along the French Mediterranean coast, where a highly significant hot spot (99%) identifies this region as the main area of recent records. The presence of high outliers is observed in the northern Adriatic.
- Northwest Europe—In the NWE, statistically significant cold spots were located further north, along the southern North Sea coasts, characterized by earlier records and suggesting the initial establishment area. In contrast, hot spots (95–99%) were concentrated along the French Atlantic coast, indicating that this area hosts the most recent occurrences and represents the current expansion front.

Overall, the temporal hot spot pattern demonstrates a non-random diffusion process, with distinct invasion nuclei and clear chronological gradients in all three basins: (i) the most spatially extensive and statistically robust clustering in the BS; (ii) a comparatively dispersed pattern in the MS, excluding the Adriatic Sea; and (iii) a more localized but strong nucleus in the NWE.

## 4. Discussion

*Rapana venosa* possesses a suite of biological and ecological traits that make it an exceptionally successful marine invader. It thrives across a wide range of habitats and tolerates low salinity, pollution, and oxygen-poor environments [204,205]. Its strong predatory capacity, particularly on economically and ecologically important bivalves, and adaptation to locally available prey contribute to significant declines in native benthic communities and disrupt local trophic structures [1]. Its dispersal potential is amplified by a planktonic larval stage that may remain in the water column for several days and tolerates broad salinity ranges, allowing larvae to be transported long distances in ballast water [206]. Once established, the species grows rapidly and early achieves sexual maturity [206]. High fecundity, coupled with successful egg deposition on hard substrates, ensures robust recruitment, while longevity exceeding 15 years maximizes reproductive output over time [7]. Remarkably, *R. venosa* can establish thriving populations even when introduced by very few individuals, demonstrating strong resilience and adaptability despite low genetic diversity [207]. Despite exhibiting traits typically associated with invasive species, its distribution is not uniform across the investigated area. This means that the species does not colonize all potentially suitable areas in a homogeneous way; rather, it appears concentrated in specific sites that are often isolated from one another. Such discontinuity may depend on several factors, including the availability of suitable habitats, environmental or geographical barriers, or the need for human-mediated vectors to facilitate its spread.

**Figure 8:**
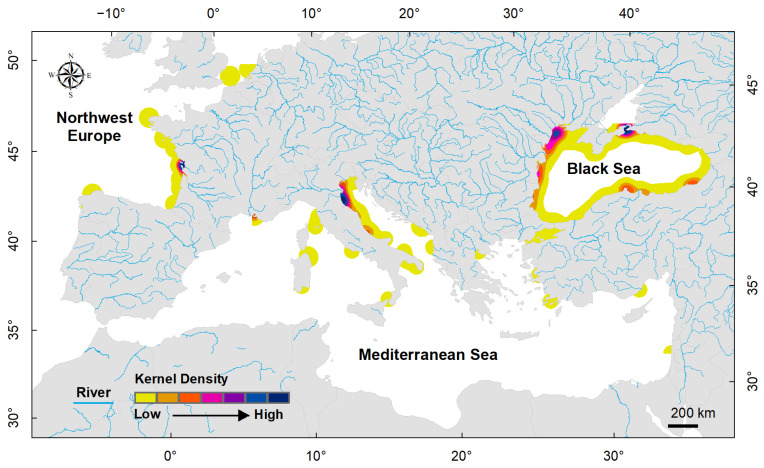
Cumulative kernel density maps showing the spatiotemporal expansion of *Rapana venosa* in all three study areas. Blue lines indicate the main rivers.

The Black Sea stands out as the most advanced and spatially expansive invasion case among the three basins examined. After a relatively short arrival phase in the northeast sector and a prolonged establishment period, the species expanded rapidly over the past two decades, being recorded along almost the entire coastline, with a stronger presence in the northwest, where highly significant hot spots are concentrated. This area is directly influenced by the outflow of the Danube, Dniester, and Dnieper rivers (Figure 8), whose large freshwater input significantly reduces local salinity levels [208]. As a result, the environmental conditions shift toward brackish values that fall within the optimal tolerance range (25 to 32) evaluated for *R. venosa*, which corresponds to that of mesohaline waters [1].

The lowered salinity supports the species’ physiological needs and enhances its reproductive success and survival. These conditions create an ecological window that facilitates the establishment and spread of *R. venosa*, accelerating its invasion across the region. The species’ success is further promoted by the absence of natural predators and by favorable environmental factors such as suitable salinity and temperature, soft-sediment habitats for burrowing, and abundant prey [209]. Overall, the configuration in the Black Sea is consistent with a mature invasion characterized by multiple aggregation zones and ongoing regional spread. This basin, with salinity about half that of the Mediterranean and relatively simple biocenoses, reflects its origin as a former freshwater lake and is characterized by the absence of animal life below ~100 m due to an expanding anoxic layer [210]. Under these conditions, the comparatively large size of *R. venosa* relative to native gastropods may have facilitated active expansion, in contrast to other European seas, where spread is typically driven by passive long-distance transport followed by local dispersal. The persistent surface outflow of low-salinity water from the Black Sea through the Sea of Marmara towards the Aegean–Mediterranean basin [211] is expected to have favored the spread of *R. venosa* into this region. However, although the species has clearly become established in the Sea of Marmara (Figure 7), the extensive and vigorous colonization that occurred in the Black Sea did not progress with the same intensity in the Aegean Sea. In this latter basin, occurrences of the species are far more sporadic, both spatially and temporally, and the populations observed so far appear to represent only the early stages of an invasion process. Such a discrepancy likely reflects differences in environmental conditions, dispersal pathways, and local ecological pressures, which may hinder establishment and limit the species’ ability to spread consistently across the Aegean, where river inputs are scarce, salinity is higher, and the fauna is more diverse, including large predatory gastropods.

In fact, while the species is euryhaline (approximately within the range of 7–30+ [1,204]) and therefore physiologically capable of withstanding broad fluctuations in salinity, its early developmental stages exhibit far narrower tolerance limits. The planktonic larvae, in particular, are highly sensitive to environmental stressors, and both elevated temperatures and high salinity—such as those characterizing large portions of the Aegean Sea—can significantly impair their survival, growth, and metamorphosis [204,212]. As a result, even though adult *R. venosa* can persist in a wide range of habitats, the combination of thermal and salinity regimes in the Aegean may constitute a substantial barrier to the establishment or expansion of self-sustaining populations.

Considering the whole Mediterranean Sea, the invasion history comprises a short arrival phase, a prolonged establishment period, and a recent expansion phase. The progressive rise in slope values over time indicates an acceleration of the invasion process, particularly after 2020, suggesting that the species remained spatially constrained for decades before undergoing a more rapid spread. The Adriatic Sea functions as the historical core area, supported by the presence of significant cold spots and persistent median centers of distribution. Even in the Adriatic Sea, as in the Black Sea, a conspicuous population nucleus can be observed near major river mouths (e.g., the Po River delta) and within lagoonal systems such as those of the northern Adriatic. In contrast, along the eastern Adriatic coast, *R. venosa* is largely absent in the northern sector, likely due to the lack of major river inputs. This spatial pattern once again highlights a certain correspondence between the distribution of *R. venosa* and the presence of brackish water environments. In these areas, the substantial freshwater input from large river systems and coastal lagoons leads to locally reduced salinity and enhanced nutrient (e.g., nitrogen and phosphorus compounds) availability—conditions that closely mirror those in which the species has successfully established and expanded in other regions [1]. However, these environmental drivers are inferred from spatial associations rather than directly tested within the present study; therefore, their role should be considered indicative rather than conclusive, as no formal analytical framework was applied to disentangle their relative contributions or to establish causal relationships. The recurring association between *R. venosa* populations and transitional waters suggests that such environments may act as ecological refugia and potential source areas, facilitating both the settlement of larvae and the persistence of adult populations. Similar considerations may be done for the Berre Lagoon (Mediterranean French coast), characterized by brackish waters, where a hotspot was detected, indicating its role as the leading edge of the ongoing expansion (Figure 7). This lagoon lies remarkably far from all other known Mediterranean invaded sites, strongly suggesting that its colonization is likely the result of anthropogenic transport rather than natural dispersal. Ultimately, this reinforces the hypothesis that brackish, nutrient-enriched zones represent key drivers in shaping the invasion dynamics of the species across different basins. The absence of self-sustaining populations at the low latitudes supports the hypothesis of thermal control acting as a barrier for the spread of the species, consistent with reports indicating that it typically survives within a temperature range of approximately 7–24 °C [1]. Prolonged exposure to elevated summer temperatures, indeed, may exceed upper tolerance thresholds of larval and juvenile stages and compromise both survival and recruitment success. Consequently, the combination of favorable salinity regimes in river-influenced areas and restrictive thermal conditions farther south helps explain why *R. venosa* forms stable populations in the northern Adriatic while failing to extend its range beyond this region. Together, these factors underscore the importance of local hydrographic and thermal regimes in shaping the species’ invasion dynamics across the Mediterranean basin. A lack of food resources can be reasonably excluded as a limiting factor. Along the Italian Adriatic coastline, indeed, eutrophic conditions are well documented [213], ensuring a continuous and abundant supply of suspended organic matter that supports dense populations of filter-feeding bivalves, such as mussels, clams, and oysters, which are the preferred food of *R. venosa* [1,214]. Moreover, the widespread presence of submerged artificial reefs, breakwaters, and other coastal defense structures provides additional hard substrate that greatly enhances larval settlement and recruitment of such organisms [215].

In Northwest Europe, the invasion history comprises a prolonged arrival phase followed by a recent establishment phase, while a distinct expansion phase has not yet emerged. The cumulative trend shows a recent increase in slope, indicating an acceleration in occurrence records but not yet a transition toward large-scale spatial spread. This suggests that the species is still consolidating its distribution rather than undergoing broad geographic expansion, as also confirmed by spatial analyses. Dispersion remained predominantly oriented along the north–south axis, maintaining a consistent NE–SW alignment from the French Atlantic coast to the Iberian Peninsula. Rather than indicating continued range expansion, the recent contraction of both axes suggests a phase of spatial consolidation and internal reorganization of the invaded area. In this context, the high Global Moran’s I value and the concentration of significant hot spots along the French Atlantic coast point to the emergence of a core invasion nucleus, likely acting as a primary source for local spread. As observed in other basins, aggregations in the Atlantic region also tend to occur near major river mouths—such as the Gironde estuary—suggesting that transitional or nutrient-rich environments may play a facilitative role in supporting persistent populations and promoting localized spread.

## 5. Conclusions

The invasion dynamics of *Rapana venosa* show basin-specific trajectories across the Black Sea, the Mediterranean Sea, and Northwest Europe, reflecting differences in invasion stages and spatial patterns.

The Black Sea hosts a mature and spatially extensive invasion, the Mediterranean Sea shows a recent and ongoing expansion accompanied by changes in distributional orientation, and Northwest Europe is characterized by a more localized distribution that is still consolidating. These differences highlight the need for monitoring and management approaches tailored to regional invasion dynamics.

The largest and most persistent populations of *R. venosa* are generally associated with major river mouths, where reduced salinity and high nutrient availability may favor settlement and recruitment. These areas therefore represent strategic focal points for monitoring efforts and for the development of targeted management actions. Focusing surveillance efforts in these zones may improve the early detection of newly established populations and support actions aimed at limiting the further spread and establishment of this invasive species.

## Figures and Tables

**Table 1:** Analyses, spatial and temporal indicators, ecological interpretation, methods, and scales (modified from Perzia et al. [15]). For the analyses, only the first records per 0.05° lat/long grid cell were considered.

Analysis/ Indicator Name	Tools	Spatial Scale	Time Scale	Ecological Meaning
Temporal and spatial–temporal pattern				
Occurrence Increase rate	Evaluation of the slopes of the cumulative occurrence curve using the least squares method	Global	BS: 1947–1959; 1960–2003; 2004–2025 MS: 1973–1982; 1983–2019; 2020–2025 NWE: 1997–2019; 2020–2025	Identification of the invasion phases; rate of specimen increases; expansion areas
Density hotspots	Kernel density BS: distance radius = 87 km MS: distance radius = 83 km NWE: distance radius = 68 km	Global	BS: 1947–1959; 1947–1983; 1947–2003; 1947–2025 MS: 1973–1982; 1973–2001; 1973–2019; 1973–2025 NWE: 1997–2004; 1997–2011; 1997–2019; 1997–2025	Nuclei of record aggregation; space–time occurrence density increase; occurrence of persistent areas; highest density areas
Key characteristics of distribution				
Center of gravity	Central tendency (median center)	Global	BS: 1947–1959; 1960–2003; 2004–2025 MS: 1973–1982; 1983–2019; 2020–2025 NWE: 1997–2003; 2005–2019; 2020–2025	Species concentration center and its change over time
Directional Dispersion	XStdDist, YStdDist; (km) Standard deviational ellipse (1 standard deviation)	Global		Species distribution in X and Y directions
Directional trends	Rotation (°) Standard deviational ellipse (1 standard deviation)	Global		Directional trend of species dispersion
Aggregation patterns and spatial structure				
Spatial autocorrelation for a series of increasing distances	Incremental spatial autocorrelation (ISA) Number of distance bands = 10	Global	All years	The distances where the clustering spatial processes are most pronounced. Distribution pattern: dispersion vs. random vs. clustering. Change in the spatial pattern over time
Statistically significant hot spots and cold spots	Getis–Ord Gi* (GOG*) BS: distance band = 87 km MS: distance band = 473 km NWE: distance band = 165 km	Local	All years	Initial and current direction of spread and identification of dispersion/settle areas
Spatial outliers	Anselin local Moran’s I (AMI) BS: distance band = 87 km MS: distance band = 473 km NWE: distance band = 165 km	Local	All years	Presence of recent records within clusters of older records (and vice versa)

## Data Availability

The original contributions presented in this study are included in the article and the Supplementary Materials. Further inquiries can be directed to the corresponding author.

## Supplementary Materials

The following supporting information can be downloaded at: https://www.mdpi.com/article/10.3390/biology15131012/s1, Table S1: *Rapana venosa* occurrence database.

## References

[1] Mann R., Occhipinti A., Harding J.M. (2004). Alien Species Alert: Rapana venosa (Veined Whelk).

[2] Drapkin E. (1963). Effect of *Rapana bezoar* Linné (Mollusca, Muricidae) on the Black Sea fauna. Dokl. Akad. Nauk SSSR.

[3] Chukhchin V.D. (1984). Ecology of the Gastropod Mollusks of the Black Sea.

[4] Aydın M., Düzgüneş E., Karadurmuş U. (2016). Rapa whelk (*Rapana venosa* Valenciennes, 1846) fishery along the Turkish coast of the Black Sea. J. Aquac. Eng. Fish. Res..

[5] Danilov C.S., Tiganov G., Anton E., Nenciu M.I., Nita V.N., Cristea V. (2018). *Rapana venosa*–New Exploitable Resource at the Romanian Black Sea Coast. Sci. Pap. Ser. D Anim. Sci..

[6] Düzgüneş E., Kasapoğlu N., Şahin A., Sağlam H. (2010). Responses to the invasive species in the Black Sea. Proceedings of the International Conference on Biodiversity of the Aquatic Environment Towards a Diverse and Sustainable World.

[7] Harding J.M., Mann R. (2016). Habitat disturbance combined with life history traits facilitate establishment of *Rapana venosa* in the Chesapeake Bay. J. Shellfish Res..

[8] Sánchez Acosta M., Góngora N., Antuña D., Correa P., Chiesa E., Brugnoli E., Muniz P. (2024). An update of the invasion status of *Rapana venosa* (Mollusca: Gastropoda) in the Río de la Plata estuary. Mar. Fish. Sci. (MAFIS).

[9] Lercari D., Bergamino L. (2011). Impacts of two invasive mollusks, *Rapana venosa* (Gastropoda) and *Corbicula fluminea* (Bivalvia), on the food web structure of the Río de la Plata estuary and nearshore oceanic ecosystem. Biol. Invasions.

[10] Falautano M., Perzia P., Castriota L. (2020). First record of the Lessepsian fish *Parexocoetus mento* in Italian waters and GIS–based spatial and temporal distribution in Mediterranean Sea. J. Mar. Biol. Assoc. U. K..

[11] Perzia P., Spinelli A., Interdonato F., Castriota L. (2022). Ecological indicators from spatial statistics to describe the Atlantic fangtooth moray distribution in Mediterranean Sea. Trans. GIS.

[12] Castriota L., Falautano M., Maggio T., Perzia P. (2022). The Blue Swimming Crab *Portunus segnis* in the Mediterranean Sea: Invasion Paths, Impacts and Management Measures. Biology.

[13] Castriota L., Falautano M., Perzia P. (2024). When Nature Requires a Resource to Be Used—The Case of *Callinectes sapidus*: Distribution, Aggregation Patterns, and Spatial Structure in Northwest Europe, the Mediterranean Sea, and Adjacent Waters. Biology.

[14] Castriota L., Falautano M., Maggio T., Perzia P. (2025). Exploring the Enigmatic Spread and Spatial Dynamics of *Bursatella leachii* in the Mediterranean Sea. Biology.

[15] Perzia P., Zampardi S., Maggio T., Falautano M., Castriota L. (2026). The Alien Jellyfish *Cassiopea andromeda* in the Mediterranean Sea: Invasion Dynamics and Management Strategies. Oceans.

[16] Lipej L., Mavric B., Paliska D. (2013). New northernmost record of the blunthead pufferfish, *Sphoeroides pachygaster* (Osteichthyes: Tetraodontidae) in the Mediterranean Sea. Ann. Ser. Hist. Nat..

[17] ESRI Kernel Density Analysis. https://pro.arcgis.com/en/pro-app/latest/tool-reference/spatial-analyst/kernel-density.htm.

[18] ESRI Median Center. https://pro.arcgis.com/en/pro-app/latest/tool-reference/spatial-statistics/median-center.htm.

[19] ESRI Directional Distribution (Standard Deviational Ellipse). https://pro.arcgis.com/en/pro-app/latest/tool-reference/spatial-statistics/directional-distribution.htm.

[20] ESRI Incremental Spatial Autocorrelation. https://pro.arcgis.com/en/pro-app/latest/tool-reference/spatial-statistics/incremental-spatial-autocorrelation.htm.

[21] ESRI Hot Spot Analysis (Getis–Ord Gi*). https://pro.arcgis.com/en/pro-app/latest/tool-reference/spatial-statistics/hot-spot-analysis.htm.

[22] ESRI Cluster and Outlier Analysis (Anselin Local Moran’s I). https://pro.arcgis.com/en/pro-app/latest/tool-reference/spatial-statistics/cluster-and-outlier-analysis-anselin-local-moran-s.htm.

[23] Abaza V., Dumitrache C., Dumitrescu E. (2010). Structure and Distribution of the Main Molluscs from the Romanian Marine Areas Designated for Their Growth and Exploitation. Cercet. Mar.-Rech. Mar..

[24] Albayrak S. (2003). On the Mollusca Fauna of the Black Sea near Istanbul. Zool. Middle East.

[25] Albayrak S., Balkıs N. (1996). Benthic Prosobranch Gastropods of the Bosphorus. İstanb. Üniv. Fen Fak. Biyol. Derg..

[26] Alkan N., Alkan A. (2023). Elemental Compositions of *Rapana venosa* (Mollusca: Muricidae) from the Eastern Black Sea Region of Turkey: Toxicology Health Risk Assessment. Anal. Lett..

[27] Alparslan M., Özalp H.B., Sağır-Odabaşı S. (2006). An Invader-Economical Gastropod *Rapana venosa* (Valenciennes, 1846) in Çanakkale and Adjacent Regions. Ege J. Fish. Aquat. Sci..

[28] Altınağaç U., Ayaz A., Kara A. (2004). A Preliminary Study on the Whelk Fisheries (*Rapana venosa*) Using Liftnets of Various Sizes. Ege J. Fish. Aquat. Sci..

[29] Altuğ G., Güler N. (2002). Determination of Indicator Bacteria, *Salmonella* spp. and Heavy Metals in Sea Snails (*Rapana venosa*) from the Northern Sea of Marmara. Turk. J. Fish. Aquat. Sci..

[30] (1990). Note & Notizie. La Conchiglia.

[31] Arbuatti A., Di Serafino A., Lucidi P. (2024). Long-Term Ecosystem Monitoring Along the Trabocchi Coast (Chieti, Italy): Insights from Underwater Visual Surveys (2011–2024). Animals.

[32] Astruch P., Schohn T., André F., Belloni B., Boudouresque C.-F., Lejeusne C., Mayot N., Marchessaux G., Thibaut T., Zibrowius H. (2025). Chaotic and Long-Term Trends in Berre Lagoon (Provence, France): A Shift Towards Alien-Dominated Assemblages?. Mediterr. Mar. Sci..

[33] Aydın M., Karadurmuş U. (2013). Karadeniz’de *Rapana* (*Rapana venosa*) Avcılığı. 17. Ulusal Su Ürünleri Sempozyumu.

[34] Baki B., Kalma M., Yücel Ș. (2010). Meat Yield of *Rapana thomasiana* Gross, 1861 Caught in Middle Black Sea Region (Sinop–Turkey). Sci. New Technol..

[35] Bañón R., Otero Mascato J.A. (2014). Nuevas Citas de *Rapana venosa* en Aguas de Galicia. Not. SEM.

[36] Bañón R., Fariña J., de Carlos A. (2023). New Data on Exotic Muricid Species from Spain Based on Integrative Taxonomy. Diversity.

[37] Başusta N. A study on the age determination using vertical shell cutting method of rapa whelk (*Rapana venosa*). Proceedings of the 2nd International Baku Conference on Scientific Research.

[38] Başusta N., Dürrani Ö., Bat L., Başusta A., Dağtekin M., Seyhan K. (2025). Modelling Growth and Population Parameters of *Rapana venosa* (Muricidae) in the Black Sea Using Statolith-Based Ageing. Fish. Sci..

[39] Bat L., Gonlaigiir G., Andae M., Öztürk M., Oztürk M. (2000). Heavy Metal Concentrations in the Sea Snail *Rapana venosa* from Sinop Coasts of the Black Sea. J. Black Sea/Mediterr. Environ..

[40] Bilecik N. (1975). La Répartition de *Rapana thomasiana thomasiana* sur le Littoral Turc de la Mer Noire. Rapp. Comm. Int. Mer. Méditerr..

[41] Boltacheva N.A., Revkov N.K., Bondarenko L.V., Kolesnikova E.A., Timofeev V.A., Kopiy V.G., Gaevskaya A.V. (2016). Taxonomic Composition of Macrozoobenthos in Karkinitskiy Bay (Black Sea) in early XXI century. Morskie Biologicheskie Issledovaniya: Dostizheniya i Perspektivy: V 3-kh t.: Sb. Materialov Vseros. Nauch.-Prakt. Konf. s Mezhdunar. Uchastiem, Priuroch. k 145-Letiyu Sevastopol’skoi Biologicheskoi stantsii (Sevastopol, 19–24 September 2016).

[42] Bombace G., Fabi G., Fiorentini L., Speranza S. (1994). Analysis of the Efficacy of Artificial Reefs located in Five Different Areas of the Adriatic Sea. Bull. Mar. Sci..

[43] Bondarev I. (2024). Peculiarities of Population Structure and Biocenotic Relationships of *Rapana venosa* (Valenciennes, 1846) (Gastropoda, Muricidae) in the Donuzlav Bay (the Black Sea). Mar. Biol. J..

[44] Bondarev I.P. (2013). Ecomorphological Analyses of Marine Mollusks’ Shell Thickness of *Rapana venosa* (Valenciennes, 1846) (Gastropoda: Muricidae). Int. J. Mar. Sci..

[45] Bondarev I.P. (2022). *Rapana venosa* (Valenciennes, 1846) in the Donuzlav Bay and Adjacent Black Sea Area. Ruthenica.

[46] Bouget J.F., Camus P., Joly J.P. (2001). Ocinebrellus inornatus (Recluz, 1851), Rapana venosa (Valenciennes, 1846): Deux Nouveaux Gastéropodes Introduits dans la Baie de Quiberon. Contrat SRC Bretagne Sud/Ifremer n°01/2.210.261.

[47] Bracchetti L., Capriotti M. (2021). Le formazioni a Reef della Costa Picena. Studi Costieri.

[48] Brustur T., Bălan S.V., Briceag A. (2021). Rapa whelk (*Rapana venosa*) Trails from the Black Sea Romanian Inner Shelf (SE Mangalia). Geo-Eco-Marina.

[49] Camus P. (2001). Un bien discret et redoutable prédateur de coquillages, l’exotique globe-trotter: *Rapana venosa*. La Vigie.

[50] Castellazzi M., Savini D., Occhipinti Ambrogi A. (2007). Shell morphotypes of the invasive gastropod *Rapana venosa* in the Northern Adriatic Sea. Boll. Malacol..

[51] Celik M., Çulha S.T., Culha M., Yildiz H., Acarli S., Celik I., Celik P. (2014). Comparative study on biochemical composition of some edible marine molluscs at Canakkale coasts, Turkey. Indian J. Geo Mar. Sci..

[52] Celik O., Samsun O. (1996). Investigation of the Catch Amount and the Catch Composition of Dredges with Various Design Features. Su Urun. Derg..

[53] Cesari P., Pellizzato M. (1985). Insediamento nella Laguna di Venezia e distribuzione adriatica di *Rapana venosa* (Valenciennes). Lav. Soc. Ven. Sc. Nat..

[54] Chandler E.A., McDowell J.R., Graves J.E. (2008). Genetically Monomorphic Invasive Populations of the Rapa Whelk, *Rapana venosa*. Mol. Ecol..

[55] Chiaro Quotidiano. https://chiaroquotidiano.it/2024/07/07/specie-aliene-in-adriatico-la-rapana-venosa-sempre-piu-diffusa-anche-nel-mare-abruzzese/.

[56] Chukhchin V.D. (1961). Rapana (*Rapana bezoar* L.) on the Gudauta Oyster Bank. Tr. Sevastopol. Biol. Stantsii.

[57] Cosentino A., Giacobbe S., Potoschi A. (2009). The CSI of the Faro Coastal Lake (Messina): A Natural Observatory for the Incoming of Marine Alien Species. Biol. Mar. Mediterr..

[58] Cristo B., Rojch L. (2009). Un Esercito di “Mostri” Popola Il Mare. Arselle Filippine e Gasteropodi Giapponesi Nel Golfo Olbiese.

[59] Crocetta F. (2011). Marine alien mollusca in the Gulf of Trieste and neighbouring areas: A critical review and state of knowledge (updated in 2011). Acta Adriat..

[60] Crocetta F., Soppelsa O. (2006). Primi ritrovamenti di *Rapana venosa* per alcune lagune costiere italiane. Atti Mus. Civ. Stor. Nat. Trieste.

[61] Cucaz M. (1983). *Rapana venosa* vivente nel Golfo di Trieste. Boll. Malacol..

[62] Çulha M., Bat L., Doğan A., Dağlı E. (2009). Ecology and Distribution of the Veined Rapa Whelk *Rapana venosa* in Sinop Peninsula. J. Anim. Vet. Adv..

[63] Dağtekin M. (2023). The Invasive Mollusk *Rapana venosa* in the Mid-Southern Black Sea: Distribution, Growth, and Stock Structure. Acta Ichthyol. Piscat..

[64] Danilov C.S., Nicolaev S., Anton E., Țiganov G., Nicolaev A.D., Păun C.V., Maximov V. (2019). Dynamics of the Fishing Capacities Used for the Mechanized Exploitation of the Gastropod *Rapana venosa* at the Romanian Coast (2013–2017). Cercet. Mar.–Rech. Mar..

[65] De Min R., Vio E. (1997). Molluschi Conchiferi del Litorale Sloveno. Annales.

[66] Delongueville C., Scaillet R. (2007). Les espèces invasives de mollusques en Méditerranée. Novapex.

[67] Delongueville C., Scaillet R. (2013). *Rapana venosa* (Valenciennes, 1846) en Mer de Marmara. Novapex.

[68] Delongueville C., Scaillet R. (2022). Le fabuleux voyage de *Rapana venosa* (Valenciennes, 1846) de l’Asie de l’Est à la Mer du Nord et aux Amériques. Novapex.

[69] Demirci G. (2005). Studies of Mollusca Fauna in Mid Black Sea, Turkiye. Fırat Üniv. Fen. Ve Müh. Bil. Der.

[70] Doneddu M. (2011). Molluschi alloctoni rinvenuti lungo le coste del comune di Olbia (Sardegna nord-orientale): Rassegna dei dati disponibili. Not. SIM.

[71] Réseau DORIS (2021). Le Rapana Veiné (*Rapana venosa*) en Métropole. DORIS Forum. https://doris.ffessm.fr/Forum/ReseauDORIS-Le-rapana-veine-Rapana-venosa-en-metropole-47044.

[72] Düzgüneş E., Unsal S., Feyzioğlu A.M. (1992). Assessment of Sea Snail (Rapana thomasiana Gross, 1861) Stocks in the Eastern Black Sea.

[73] Düzgüneş E., Feyzioğlu A.M. (1994). Trabzon Sahil Şeridinde Yaşayan Deniz Salyangozunun Rapana thomasiana Gross. 1861 Populasyon ve Büyüme Özelliklerinin Araştırılması. Ege Üniv. Fen Fak. Derg..

[74] Engl W. (1995). Specie prevalentemente lessepsiane attestate lungo le coste Turche. Boll. Malacol..

[75] Erik G., Dağtekin M. (2021). Effect of fishery closure on discard composition of Rapa Whelk *Rapana venosa* (Valenciennes, 1846) beam trawl fisheries in the Black Sea. Turk. J. Fish. Aquat. Sci..

[76] Eryasar A.R., Ceylan Y., Dalgic G., Yesilcicek T. (2018). By-catch in the commercial beam trawl fishery for rapa whelk in the Black Sea. Mediterr. Mar. Sci..

[77] Florio M., Breber P., Scirocco T., Specchiulli A., Cilenti L., Lumare L. (2008). Exotic species in Lesina and Varano lakes: New guest in Lesina and Varano lakes (Gargano National Park, Italy). Trans. Waters Bull..

[78] Georgiev A.P.D., Nikolov A.P.G., Kalcheva A.P.S. (2020). Influence of rapana (*Rapana venosa*) on the condition of the mussel populations in the water area of the town of Primorsko. Proceedings Of University of Ruse–2020.

[79] Ghisotti F. (1974). *Rapana venosa* (Valenciennes), nuova ospite Adriatica?. Conchiglie.

[80] Giacobbe S., De Domenico F., Iarrera S.P.M., Mangano M.C., Porporato E., Spanò N. (2010). Alien molluscs and crustacean decapods in the Strait of Messina (Central Mediterranean Sea). Rapp. Comm. Int. Mer Méditerr..

[81] Giannuzzi-Savelli R., Pusateri F., Palmeri A., Ebreo C. (2003). Atlante Delle Conchiglie Marine del Mediterraneo–Atlas of the Mediterranean Sea Shells.

[82] Golikov A., Scarlato O.A., Starobogatov J. (1972). Opredeliteli Fauny Chernogo i Azovskogo Morei.

[83] Gomoiu M.-T. (1972). Some ecologic data on the gastropod *Rapana thomasiana* Crosse along the Romanian Black Sea shore. Cercet. Mar.–Rech. Mar..

[84] Goncharov A.D. (1977). *Rapana* from the northwestern coast of the Black Sea. Hydrobiol. J..

[85] Gönener S., Özsandıkçı U. (2017). Density distribution and some biological properties of veined rapa whelk *Rapana venosa* populations in the south-central Black Sea. Cah. Biol. Mar..

[86] Goulletquer P. (2016). Guide des Organismes Exotiques Marins.

[87] Govorin I.A. (2013). Use of the Morphometric Characteristics of Empty Shells of *Rapana thomasiana* (Mollusca, Gastropoda), Found in After-Storm Ejects, for the Determination of Lifetime Mass Indices of Mollusks. Vestn. Zool..

[88] Govorin I.A. (2018). The Predatory Marine Gastropod *Rapana venosa* (Valenciennes, 1846) in Northwestern Black Sea: Morphometric Variations, Imposex Appearance and Biphallia Phenomenon. Molluscs.

[89] Govorin I.A., Kurakin A.P. (2005). Finding of *Rapana thomasiana* in the near Danube Delta area of the Black Sea [Nakhodka rapany Rapana thomasiana v pridunajskom rajonie severozapadnoj chasti Chernogo moria]. Ecol. Morya.

[90] Grossu A.V., Lupu D. (1964). The Presence of *Rapana bezoar* Opposite the Rumanian Black Sea Shores (Muricidae). Arch. Molluskenkd..

[91] Grossu A. (1970). Two Species Recently Discovered Invading the Black Sea. Sea Shore.

[92] Hubenov Z., Kenderov L., Pandourski I. (2015). Invertebrate Animals (Metazoa: Invertebrata) of the Atanasovsko Lake, Bulgaria. Hist. Nat. Bulg..

[93] Hulak B.S., Leonchyk Y.Y., Chashchyn O.K. (2022). The Main Biological Parameters of Rapa Whelk *Rapana venosa* Population in the Northwestern Section of the Black Sea. Hydrobiol. J..

[94] Hulak B.S., Leonchyk Y.Y., Snigiriev S.M., Chashchyn O.K. (2024). State of the Commercial Stock of Rapa Whelk *Rapana venosa* in the Coastal Zone of the Northwestern Section of the Black Sea. Hydrobiol. J..

[95] ICES (2001). Report of the Working Group on Introductions and Transfers of Marine Organisms (WGITMO).

[96] ICES (2012). Report of the ICES Working Group on Introduction and Transfers of Marine Organisms (WGITMO).

[97] ICES (2004). Report of the Working Group on Introductions and Transfers of Marine Organisms (WGITMO).

[98] ICES (2005). Report of the Working Group on Introductions and Transfers of Marine Organisms (WGITMO), by Correspondence.

[99] ICES (2006). Working Group on Introductions and Transfers of Marine Organisms (WGITMO).

[100] ICES (2007). Report of the Working Group on Introductions and Transfers of Marine Organisms (WGITMO).

[101] ICES (2010). Report of the ICES Working Group on Introductions and Transfers of Marine Organisms (WGITMO).

[102] ICES (2013). Report of the ICES Working Group on Introduction and Transfers of Marine Organisms (WGITMO).

[103] Iliescu M., Rădulescu I. (1968). Note Concerning the Gastropod *Rapana bezoar* L. on the Romanian Shore. Bul. Cercet. Piscic..

[104] iNaturalist. https://www.inaturalist.org/observations?taxon_id=370913.

[105] Ivanov A.I. (1961). Some Data on the Quantitative Distribution of *Rapana bezoar* (L.) in the Eastern Part of the Black Sea and in the Kerch Strait and the Decrease in Its Size. Dokl. Akad. Nauk SSSR.

[106] Ivanov A.I. (1968). Changes in the abundance of *Rapana* in the Kerch Strait in 1958-1965. Hydrobiol. J..

[107] Ivanov D.A., Sinegub I.A. (2007). Transformation of Biocenosis of the Kerch Strait After the Introduction of the Predatory Mollusk *Rapana thomasiana* and the Bivalves *Mya arenaria* and *Cunearca cornea*. In Contemporary Problems of Ecology in the Azov-Black Sea Region. Proceedings of the III International Conference, 10–11 October 2007.

[108] Ivanova P.P., Trayanova A.T., Stefanova K.B., Stefanova E., Raykov V.S., Doncheva V.G. (2017). Population Status of Some Alien Species in Varna Bay, Bulgarian Black Sea Coast (2015–2016). Acta Zool. Bulg..

[109] Joly J.-P., Bouget J.-F., Hirata T. (2002). Le Gastéropode Prédateur Rapana venosa: Point sur les Connaissances et Observations en Laboratoire.

[110] Kaneva-Abadjieva V. (1958). A New Harmful Snail on Our Coast of Black Sea. Nature.

[111] Kasapoğlu N. (2021). Population Structure and Shell Dimension of the Invasive Veined Whelk (*Rapana venosa*). J. Fish..

[112] Katsanevakis S., Poursanidis D., Hoffman R., Rizgalla J., Rothman B.-S.S., Levitt-Barmats Y., Hadjioannou L., Trkov D., Garmendia J.M., Joxe M. (2020). Unpublished Mediterranean records of marine alien and cryptogenic species. BioInvasions Rec..

[113] Kaykac M.H., Gümüş A., Zengin M., Süer S., Rüzgar M., Van A., Tosunoğlu Z. (2018). Effect of Steel Wire and Claw Size on the Sea Snail (*Rapana venosa*) Catch in a Black Sea Beam Trawl Fishery. Turk. J. Vet. Anim. Sci..

[114] Kerckhof F., Vink R.J., Nieweg D.C., Post J.N. (2006). The Veined Whelk *Rapana venosa* Has Reached the North Sea. Aquat. Invasions.

[115] Khoroshutina O., Pierce G.J., Lishchenko F. (2024). Statoliths of *Rapana venosa* (Valenciennes, 1846): Microstructure, application for age determination and growth modelling. Reg. Stud. Mar. Sci..

[116] Kinzelbach R. (1986). New Records of Thomas’ Rapa Whelk *Rapana venosa* from the Black Sea and Mannara Sea. Zool. Middle East.

[117] Kiseleva G.A., Konovalov V.S., Lapchenko A.A., Kolova K.A. (2009). Species Composition and Dynamics of the Macrozoobenthos in Algal Associations of the Karadag Nature Reserve. Ecosyst. Their Optim. Prot..

[118] Kolyuchkina G.A., Syomin V.L., Spiridonov V.A., Zalota A.K., Biryukova S.V., Basin A.B., Simakova U.V., Nabozhenko M.V. (2019). The Resilience of Macrozoobenthos of Boreal Coastal Lagoons to Non-Indigenous Species Invasion: A Case Study of Taman Bay (Sea of Azov). Reg. Stud. Mar. Sci..

[119] Konsulova T.H. (1992). *Mytilus galloprovincialis* Natural Resources Along the Northern Bulgarian Black Sea Coast in Relation to *Rapana thomasiana* Distribution. Trudy Instituta Oceanologii.

[120] Kosarev A.N., Kostianoy A.G., Shiganova T.A. (2007). The Sea of Azov. The Black Sea Environment.

[121] Kos’yan A.R. (2013). Comparative Analysis of *Rapana venosa* from Different Biotopes of the Black Sea Based on Morphological Characteristics. Oceanology.

[122] Kosyan A.R., Kucheruk N.V., Flint M.V. (2012). Role of Bivalve Mollusks in the Sediment Balance of the Anapa Bay Bar. Oceanology.

[123] Kousteni V., Bakiu R., Benhmida A., Crocetta F., Di Martino V., Dogrammatzi A., Doumpas N., Durmishaj S., Giovos I., Trkov D. (2019). New Mediterranean Biodiversity Records (April 2019). Mediterr. Mar. Sci..

[124] Koutsoubas D., Voultsiadou-Koukoura E. (1990). The Occurrence of *Rapana venosa* in the Aegean Sea. Boll. Malacol..

[125] Kovtun O.O., Toptikov V.A., Totsky V.M. (2014). Comparative Morphological Characteristics of *Rapana venosa* from Different Water Areas of the Northern Black Sea. Vistn. ONU.

[126] Koyun S., Ozcan T. (2018). Invasive Macroinvertebrate Species Monitored on the Turkish Coast Between 2014 and 2015. Turk. J. Water Sci. Manag..

[127] Kurakin A.P., Govorin I.A. (2011). Rate of *Mytilus galloprovincialis* Consumption by *Rapana venosa* in the Northwestern Black Sea. Hydrobiol. J..

[128] Lazzari G. (1982). Un “caso di coscienza”: Si può uccidere un “enfant terrible”?. Nat. Mont..

[129] L’Étang Nouveau. https://letangnouveau.wordpress.com/2015/11/09/rapana-venosa-ou-les-arrivees-plus-ou-moins-bienvenues/.

[130] Le Duff M., Cadiou S., Grall J. (2013). *Rapana venosa* (Valenciennes, 1846), une nouvelle trouvaille inquiétante. An. Aod–Les Cah. Nat. De L’observatoire Mar..

[131] Le Fur I., Deborde J., Guesdon S., Piraud A., Gautier E., Gueux A., Geairon P., Gervais H., Grizon J., Pépin J.-F. (2021). 2021-Qualité du Milieu Marin Littoral. Bulletin de la Surveillance 2020. Départements de Charente-Maritime et de Vendée (sud).

[132] Makarov M.V. (2016). Size-weight Structure of *Rapana venosa* (Mollusca, Gastropoda) near Crimean coast in 2008. Biodiversity of Caucasus and South of Russia. Proceeding of XVIII International Conference.

[133] Mel P. (1976). Sulla presenza di *Rapana venosa* (Valenciennes) e di *Charonia variegata sequenzae* nell’Alto Adriatico. Conchiglie.

[134] Micu S., Kelemen B., Mustata G. (2008). Current distribution and shell morphotypes of *Rapana venosa* (Valenciennes, 1846) in the Agigea 4 m littoral. Analele Ştiinţifice Ale Univ. “Al. I. Cuza” Iaşi Ser. Biol. Anim..

[135] Mienis H.K. (2004). New Data Concerning the Presence of Lessepsian and Other Indo-Pacific Migrants among Mediterranean Molluscs, with Emphasis on Israel. Turk. J. Aquat. Life.

[136] Milyutin D.M., Vilkova O.Y. (2006). Black Sea Invasive Mollusks *Rapana* and *Anadara*: Current State of Populations and Stock Dynamics. Rybn. Khoz..

[137] Morhun H., Son M., Kovtun O., Utevsky S. (2021). Morphological and Molecular Studies of the Rapa Whelk *Rapana venosa* (Neogastropoda, Muricidae) from Odesa Bay. Zoodiversity.

[138] Mutlu E., Kideys A.E., Şahin F., Erik G., Aksu H., Erdem E., Karayücel S., Bat L. (2022). Population dynamics and ecology of the invasive veined rapa whelk, *Rapana venosa* in the southern Black Sea. Estuar. Coast. Shelf Sci..

[139] Namiesnik J., Szefer P., Moncheva S., Ham K.S., Kang S.G., Arancibia-Avila P., Toledo F., Goshev I., Gorinstein S. (2012). Characterization of *Rapana thomasiana* as an Indicator of Environmental Quality on the Bulgarian Black Sea Coast. Environ. Technol..

[140] Naturamediterraneo. https://www.naturamediterraneo.com/forum/topic.asp?TOPIC_ID=242522.

[141] Nieweg D.C., Post J.N.J., Vink R.J. (2005). *Rapana venosa* (Gastropoda: Muricidae): A New Invasive Species in the North Sea. Deinsea.

[142] Önel E.S., Türkmen M., Kalıpcı E. (2024). Hazard Analysis and Human Health Risk Assessment in *Mytilus galloprovincialis* and *Rapana venosa* from the Turkish Black Sea Coast via Multivariate Analysis. Biol. Trace Elem. Res..

[143] Öztürk B., Zibrowius H. (1991). Ongoing modification of the Mediterranean marine fauna and flora by the establishment of exotic species. Mésogée.

[144] Paolini P. (1987). Nuova segnalazione di *Rapana venosa* nell’Alto Adriatico. Quad. Mus. Stor. Nat. Livorno.

[145] Pepin J.F., Curd A., Massé C., Bouquet A.L., Daffe C., Droual G., Dubreuil C., Nunes F., Roche E., Antajan E. (2024). Detection of Two Non-Indigenous Marine Species in the Bay of Biscay Threatening Coastal Biodiversity and Shellfish Farming via Environmental DNA and Molecular Approaches. Proceedings of ISOBAY 18–XVIII International Symposium on Oceanography of the Bay of Biscay, 5–7 June 2024.

[146] Pereladov M.V. (2013). Modern Status and Biological Aspects of the Veined Rapa Whelk *Rapana venosa* in the Northeast Black Sea. Commer. Species Their Biol. Proc. VNIRO.

[147] Perk F. (2012). De geaderde stekelhoren *Rapana venosa* al twee keer op het Nederlandse strand gevonden. Spirula.

[148] Petrova E., Mihneva V., Stoykov S., Tserkova F., Valchev S., Penchev F. (2022). Biological Monitoring of the Landed Rapana Catch by the Bulgarian Fishery Fleet, 2020–2021.

[149] Petrova E., Mihneva V., Stoykov S., Klisarova D., Uzunova S., Dineva S., Gerdzhikov D. (2017). Environmental status and dynamics of shellfish resources-veined rapa whelk and black mussel in the western Black Sea during 2015–2016. Proceedings of the Institute of Fishing Resources, Varna.

[150] Petrova E., Mihneva V., Tserkova F., Stoykov S., Valchev S., Penchev P. (2019). Biological Monitoring of the Landed Rapana Catch by the Bulgarian Fishing Fleet for the 2nd Quarter of 2019.

[151] Petrova E., Tserkova F., Mihneva V., Stoykov S., Valchev S., Penchev P. (2020). Fish Bycatch Rate in the *Rapana venosa* Fishery in Bulgarian Black Sea Waters, with Special Emphasis on *Scophthalmus maximus*. Acta Zool. Bulg..

[152] Pirkova A.V., Ladygina L.V., Shchurov S.V. (2023). Feeding Intensity of Female and Male *Rapana venosa* (Valenciennes, 1846) in the Black Sea. Vestn. Tomsk. Gos. Univ. Biol..

[153] Poggiani L., Micali P. (2018). I Molluschi del Mare di Fano e del Bacino del Metauro.

[154] Popescu-Marinescu V., Paladian G. (1971). Biological and biometric observations on *Rapana thomasiana* Crosse (Gastropoda, Muricidae). Stud. Cercet. Biol. Ser. Zool. Bucar..

[155] Popova T., Stratev D., Vashin İ., Zhelyazkov G., Valkova E., Dospatliev L. (2017). Seasonal Changes in Quality and Fatty Acid Composition of *Rapana venosa* from the Bulgarian Black Sea Coast. Turk. J. Agric. Nat. Sci..

[156] Prodanov K., Konsulova T., Tadorova V. (1995). Growth Rate of *Rapana thomasiana* Along the Bulgarian Black Sea Coast. Proceedings of the 34th CIESM Congress.

[157] Ragkousis M., Zenetos A., Ben Souissi J., Hoffman R., Ghanem R., Taşkın E., Muresan M., Karpova E., Slynko E., Dagli E. (2023). Unpublished Mediterranean and Black Sea records of marine alien, cryptogenic, and neonative species. BioInvasions Rec..

[158] Rinaldi E. (1985). *Rapana venosa* (Valenciennes) spiaggiata in notevole quantità sulla spiaggia di Rimini (FO). Boll. Malacol..

[159] Rolán E., Bañón R. (2007). Primer hallazgo de la especie invasora *Rapana venosa* y nueva información sobre *Hexaplex trunculus* en Galicia. Not. SEM.

[160] Roşioru D.M., Oros A., Coatu V., Stoica E., Negreanu-Pîrjol T. (2020). Estimation of *Rapana venosa* (Valenciennes, 1846) quality, a marine living resource from the Romanian Black Sea with bioeconomic importance. UPB Sci. Bull. Ser. B Chem. Mater. Sci..

[161] Ruci S., Kasemi D., Beqiraj S. (2014). Data on Macrozoobenthos in Rocky Areas of the Adriatic Sea of Albania in Spring Season. IMPACT Int. J. Res. Appl. Nat. Soc. Sci..

[162] Sabatini S. (1981). I molluschi: Mostra preliminare. Musei Della Città, Sez. Nat..

[163] Saenko E.M., Shaganov V.V. (2021). The Spatial Distribution and Biological Characteristics of *Rapana venosa* in the Coastal Zone of Southeastern Crimea. Probl. Fish..

[164] Saenko E.M., Marushko E.A., Bragina T.M. (2018). Status of the Veined Rapa Whelk *Rapana venosa* Population in the Northeastern Black Sea. Aquat. Bioresour. Environ..

[165] Saglam H., Duzgunes E. (2014). Biological Parameters and Feeding Behaviour of the Invasive Whelk *Rapana venosa* in the Southeastern Black Sea (Turkey). J. Coast. Life Med..

[166] Saglam H., Duzgunes E. (2007). Deposition of Egg Capsule and Larval Development of *Rapana venosa* in the Southeastern Black Sea. J. Mar. Biol. Assoc. U. K..

[167] Saglam H., Kutlu S., Bascinar S., Dagtekin M., Duzgunes E., Sahin A. (2007). Pot Fishery of *Rapana venosa* in the Southeastern Black Sea. Rapp. Comm. Int. Mer Méditerr..

[168] Saglam H., Kutlu S., Dagtekin M., Bascinar S., Sahin A., Selen H., Duzgunes E. (2015). Population Biology of *Rapana venosa* in the Southeastern Black Sea. Cah. Biol. Mar..

[169] Sağlam N.E., Sağlam C., Sağlam Y.D. (2015). Determination of some population parameters of the veined rapa whelk (*Rapana venosa*) in the Central Black Sea. J. Mar. Biol. Assoc. U. K..

[170] Sahin C., Duzgunes E., Engin S., Mutlu C., Hacimurtazaoglu N. (2005). Analysis of Age and Growth Parameters of *Rapana thomasiana*. Turk. J. Aquat. Life.

[171] Sauriau P.G., De Montaudouin X., Aubert F., Charpentier P., De Casamajor M.N., Fichet D., Guyot T., Jourde J., Le Duigou M., Massé C. (2023). Sur quelques curiosités d’histoire naturelle dans les Pertuis Charentais: Faune des invertébrés marins. Ann. Soc. Sci. Nat. Charente-Marit..

[172] Savini D., Castellazzi M., Occhipinti-Ambrogi A. (2005). Observations on Imposex in the Alien *Rapana venosa* in the Northern Adriatic Sea. Biol. Mar. Mediterr..

[173] Savini D., Castellazzi M., Favruzzo M., Occhipinti-Ambrogi A. (2004). The Alien Mollusc *Rapana venosa* in the Northern Adriatic Sea: Population Structure and Shell Morphology. Chem. Ecol..

[174] Savini D., Occhipinti-Ambrogi A., Castellazzi M. (2007). Distribution of the Alien Gastropod *Rapana venosa* in the Northern Adriatic Sea. Rapp. Comm. Int. Mer Méditerr..

[175] Sayenko E.M., Marushko E.A. (2020). Results of Rapana Monitoring in the Azov Sea in 2014–2015. Tr. Azniirkh.

[176] Sereanu V., Feraru D.L., Predeanu G., Meghea A. (2017). Microstructure and Chemical Characterization of *Rapana thomasiana* Shell. UPB Sci. Bull. Ser. C.

[177] Sereanu V., Meghea I., Vasile G.G., Mihai M. (2018). Environmental Influence on *Rapana venosa* Shell Morphotypes from the Romanian Black Sea Coast. Currents.

[178] Serrazanetti G.P., Pagliuca G., Fabbri M., Zironi E., Gazzotti T., Kindt M. (1999). Composizione lipidica di *Rapana venosa* della costa marchigiana. Biol. Mar. Mediterr..

[179] Seyhan K., Khan U., Şahin A., Terzi Y. (2022). Catch Composition of Non-Target Species Escaping Dredge Gear of *Rapana* Fisheries on the Western Black Sea Coast. Mar. Biol. Res..

[180] Seyhan K., Mazlum E.R., Emiral H., Engin S., Demirhan S. (2003). Diel Feeding Periodicity, Gastric Emptying, and Estimated Daily Food Consumption of *Rapana venosa* in the Southeastern Black Sea. Indian J. Mar. Sci..

[181] Shadrin N., Afanasova T.A. (2009). Distribution and feeding of *Rapana venosa* (Valenciennes, 1846) in water area of the Opukski nature reserve (East Crimea, the Black Sea). Mar. Ecol. J..

[182] Sinegub I.A. (2004). Macrofauna of the Upper Sublittoral Zone of Rocks in the Black Sea Near Karadag // Karadag. Hydrobiological Studies: Collected Scientific Papers for the 90th Anniversary of the Karadag Scientific Station Named After T. I. Vyazemsky and the 25th Anniversary of the Karadag Nature Reserve of the National Academy of Sciences of Ukraine–Sevastopol.

[183] Slynko E., Slynko Y.V., Rabushko V.I. (2020). Adaptive Strategy of *Rapana venosa* in the Invasive Black Sea Population. Biosyst. Divers..

[184] Smagovicz K. (1989). Polymorphism and Anomalous Shells in Juveniles of *Rapana thomasiana*. Folia Malacol..

[185] Snigirov S., Medinets V., Chichkin V., Sylantyev S. (2013). *Rapana venosa* Controls Demersal Community Structure off Zmiinyi Island (Black Sea). Aquat. Invasions.

[186] Sous la Surface de l’étang de Berre. https://souslasurfacedeletang.home.blog/2020/05/20/linstallation-du-rapana-venossa/.

[187] Stadnichenko S., Kurakin A. (2022). Feeding Intensity and Daily Mussel Consumption of *Rapana venosa* in the Northwestern Black Sea. Oceanol. Hydrobiol. Stud..

[188] Stamouli C., Akel E.H.K., Azzurro E., Bakiu R., Bas A.A., Bitar G., Boyaci Y., Cakalli M., Corsini-Foka M., Zenetos A. (2018). New Mediterranean Biodiversity Records (December 2017). Mediterr. Mar. Sci..

[189] Stark I.N. (1956). The Gudauta Oyster Bank. Priroda.

[190] Teacă A., Begu T., Gomoiu M.T. (2008). Starea ecologică a populaţiilor de *Rapana venosa* de la litoralul românesc al Mării Negre. Geo-Eco-Marina.

[191] Terreni G. (1980). Molluschi poco conosciuti dell’Arcipelago Toscano: 1—Gasteropodi. Boll. Malacol..

[192] Toptikov V.A., Totsky V.N., Alyeksyeyeva T.G., Kovtun O.A. (2014). Comparative Analysis of Adaptive Potential of *Rapana venosa* and *Mytilus galloprovincialis* Populations from the Same Biotope. Bull. ONU Ser. Biol..

[193] Trayanova A. (2016). Size-Weight Characteristics, Gender Structure and Density of *Rapana venosa* (Valenciennes, 1846) Population in Varna Bay. Union Sci..

[194] Trayanova A. (2018). Morphometric, Quantitative and Population Characteristics of *Rapana venosa* in Front of Pasha Dere. Ann. Union Sci. Varna Ser. Mar. Sci. (Oceanol.).

[195] Trigo J.E., Vieites N. (2013). Segunda cita de *Rapana venosa* para la Península Ibérica. Not. SEM.

[196] Valkanov A., Marinov T. (1964). Nachtrag zum Katalog der Bulgarischen Schwarzmeerfauna. Bull. Inst. Zool. Mus..

[197] Yeşilbudak B. (2022). Population Characteristics of *Rapana venosa* in Çamburnu Bay (Trabzon). Osman. Korkut Ata Üniv. Fen Bilim. Derg..

[198] Yildiz H., Gunduz F. (2022). Reproduction Cycles of Veined Rapa Whelk (*Rapana venosa*, Valenciennes 1846) in the Middle Black Sea (Yakakent, Samsun). Fresenius Environ. Bull..

[199] Yokeş M.B., Güçlüsoy H., Bilecenoğlu M., Çınar M.E. (2022). Ecological Effects of Rapana venosa (Veined Rapa Whelk) and Suggested Measures to be Taken. The Scientific Conference on the Impacts of the Marine Invasive Species, 21–23 October 2022.

[200] Zenetos A., Delongueville C., Scaillet R. (2024). An Overlooked Group of Citizen Scientists in Non-Indigenous Species Information: Shell Collectors and Their Contribution to Molluscan NIS Xenodiversity. Diversity.

[201] Zengin M., Süer S., Rüzgar M., Üzer U. (2023). An Evaluation of the Success and Failure of Traps Designed for *Rapana venosa* Fisheries on the Southeastern Black Sea Coast. Transylv. Rev. Syst. Ecol. Res..

[202] Zolotarev P.N., Terentyev A.S. (2012). Changes in the macrobenthic communities of the Gudauta oyster bank. Oceanology.

[203] Zolotarev P.N., Yevchenko O.V. (2010). Biology and Stock Assessment of *Rapana venosa* in the Northeastern Black Sea (1988–1994). Vopr. Rybolov..

[204] Mann R., Harding J.M. (2003). Salinity tolerance of larval Rapana venosa: Implications for dispersal and establishment of an invading predatory gastropod on the North American Atlantic coast. Biol. Bull..

[205] Munari C., Mistri M. (2011). Short-term hypoxia modulates *Rapana venosa* (Muricidae) prey preference in Adriatic lagoons. J. Exp. Mar. Biol. Ecol..

[206] Harding J.M. (2006). Growth and development of veined rapa whelk *Rapana venosa* Veligers. J. Shellfish Res..

[207] Xue D.X., Graves J., Carranza A., Sylantyev S., Snigirov S., Zhang T., Liu J.X. (2018). Successful worldwide invasion of the veined rapa whelk, *Rapana venosa*, despite a dramatic genetic bottleneck. Biol. Invasions.

[208] Mamoutos I.G., Androulidakis Y., Potiris E.I., Kolovoyiannis V., Tragou E., Zervakis V., Krestenitis Y.N. (2026). Is the exchange between the Black Sea and the Mediterranean changing over time?. Environ. Res. Commun..

[209] Bondarev I.P. (2014). Dynamics of *Rapana venosa* (Valenciennes, 1846)(Gastropoda: Muricidae) population in the Black Sea. Int. J. Mar. Sci..

[210] Neretin L.N., Volkov I.I., Rozanov A.G., Demidova T.P., Falina A.S. (2006). Biogeochemistry of the Black Sea anoxic zone with a reference to sulphur cycle. Past and Present Water Column Anoxia.

[211] Aydoğdu A., Pinardi N., Özsoy E., Danabasoglu G., Gürses Ö., Karspeck A. (2018). Circulation of the Turkish Straits System under interannual atmospheric forcing. Ocean Sci..

[212] Ban S., Zhang T., Pan H., Pan Y., Wang P., Xue D. (2014). Effects of Temperature and Salinity on the Development of Embryos and Larvae of the Veined Rapa Whelk *Rapana venosa* (Valenciennes, 1846). Chin. J. Oceanol. Limnol..

[213] Sani T., Campanelli A., Marini M., Goffredo S., Grilli F. (2026). Long-term changes in the Adriatic Sea (1971–2023): River influence, climate impacts, and biogeochemical shifts in coastal bottom waters. Estuar. Coast. Shelf Sci..

[214] Savini D., Occhipinti-Ambrogi A. (2006). Consumption rates and prey preference of the invasive gastropod *Rapana venosa* in the northern Adriatic Sea. Helgol. Mar. Res..

[215] Spagnolo A., Ferrà C. (2025). Italy’s Contribution to Artificial Reef Research: A Comprehensive Review (1970–2025). Water.

