# Supplementary material for: Spatial Patterns of the Marine Alien Gastropod Rapana venosa Invasion Across the Black Sea, Mediterranean, and Atlantic Europe
Source: Biology (Basel). 2026 Jun 25;15(13):1012. doi: 10.3390/biology15131012 (PMC13359707; doi:10.3390/biology15131012)
Supplement: Supplementary file 1 [file biology-15-01012-s001.zip › biology-4346096-supplementary.pdf]

Supplementary Materials

Table S1. *Rapana venosa* occurrences database.

SPECIES	AREA	LATITUDE	LONGITUDE	YEAR	REFERENCE
Rapana venosa	Mediterranean Sea	44.203333	12.395000	2001	[1]
Rapana venosa	Mediterranean Sea	44.203333	12.443333	2001	[1]
Rapana venosa	Mediterranean Sea	44.411667	12.395000	2001	[1]
Rapana venosa	Mediterranean Sea	44.411667	12.443333	2001	[1]
Rapana venosa	Black Sea and adjacent waters	44.705051	37.813249	1947	[2]
Rapana venosa	Black Sea and adjacent waters	41.171200	40.797300	1950	[3]
Rapana venosa	Black Sea and adjacent waters	44.580700	33.370700	1952	[3]
Rapana venosa	Black Sea and adjacent waters	44.410400	33.700100	1952	[3]
Rapana venosa	Black Sea and adjacent waters	42.658600	27.750400	1956	[3]
Rapana venosa	Black Sea and adjacent waters	43.189200	27.977900	1956	[3]
Rapana venosa	Black Sea and adjacent waters	45.495200	36.021200	1956	[3]
Rapana venosa	Black Sea and adjacent waters	41.017100	39.722300	1959	[3]
Rapana venosa	Black Sea and adjacent waters	45.403900	30.301600	1961	[3]
Rapana venosa	Black Sea and adjacent waters	45.779500	33.074400	1968	[3]
Rapana venosa	Black Sea and adjacent waters	46.070300	32.987800	1968	[3]
Rapana venosa	Black Sea and adjacent waters	41.197200	37.043600	2015	[4]
Rapana venosa	Black Sea and adjacent waters	41.149600	37.287600	2015	[4]
Rapana venosa	Black Sea and adjacent waters	41.036300	37.506500	2015	[4]
Rapana venosa	Black Sea and adjacent waters	41.067700	37.780800	2015	[4]
Rapana venosa	Black Sea and adjacent waters	40.988300	37.899300	2015	[4]
Rapana venosa	Black Sea and adjacent waters	44.293410	28.844099	2009	[5]
Rapana venosa	Black Sea and adjacent waters	44.713054	29.343991	2009	[5]
Rapana venosa	Black Sea and adjacent waters	43.752180	28.578750	2009	[5]
Rapana venosa	Black Sea and adjacent waters	43.785543	28.586205	2009	[5]
Rapana venosa	Black Sea and adjacent waters	41.440000	36.430000	1962	[6]
Rapana venosa	Black Sea and adjacent waters	40.710000	28.190000	1966	[6]
Rapana venosa	Black Sea and adjacent waters	45.004900	29.742400	2006	[23]

SPECIES	AREA	LATITUDE	LONGITUDE	YEAR	REFERENCE
Rapana venosa	Black Sea and adjacent waters	44.638500	29.160300	2006	[23]
Rapana venosa	Black Sea and adjacent waters	44.252700	28.697300	2006	[23]
Rapana venosa	Black Sea and adjacent waters	44.000000	28.692800	2006	[23]
Rapana venosa	Black Sea and adjacent waters	41.408990	28.513810	2000	[24]
Rapana venosa	Black Sea and adjacent waters	41.332160	28.792930	2000	[24]
Rapana venosa	Black Sea and adjacent waters	41.217222	29.127222	1993	[25]
Rapana venosa	Black Sea and adjacent waters	41.154900	37.170700	2016	[26]
Rapana venosa	Black Sea and adjacent waters	40.981700	38.038200	2016	[26]
Rapana venosa	Black Sea and adjacent waters	40.960100	38.708900	2016	[26]
Rapana venosa	Black Sea and adjacent waters	41.052300	39.277500	2016	[26]
Rapana venosa	Black Sea and adjacent waters	41.013400	40.354600	2016	[26]
Rapana venosa	Black Sea and adjacent waters	40.399100	26.654500	1994	[27]
Rapana venosa	Black Sea and adjacent waters	40.350200	26.676100	2004	[27]
Rapana venosa	Black Sea and adjacent waters	40.379600	26.710000	2004	[27]
Rapana venosa	Black Sea and adjacent waters	40.419800	27.054300	2006	[27]
Rapana venosa	Black Sea and adjacent waters	40.958900	39.867700	2004	[28]
Rapana venosa	Black Sea and adjacent waters	40.956400	39.934200	2004	[28]
Rapana venosa	Black Sea and adjacent waters	40.951139	28.805620	2000	[29]
Rapana venosa	Mediterranean Sea	40.942700	9.607300	1990	[30]
Rapana venosa	Mediterranean Sea	42.291944	14.473333	2011	[31]
Rapana venosa	Mediterranean Sea	42.273889	14.509722	2015	[31]
Rapana venosa	Mediterranean Sea	43.454879	5.154956	2023	[32]
Rapana venosa	Mediterranean Sea	43.471399	5.131809	2023	[32]
Rapana venosa	Mediterranean Sea	43.489024	5.122416	2023	[32]
Rapana venosa	Mediterranean Sea	43.505933	5.112674	2023	[32]
Rapana venosa	Mediterranean Sea	43.511303	5.114126	2023	[32]
Rapana venosa	Mediterranean Sea	43.524694	5.110202	2023	[32]
Rapana venosa	Mediterranean Sea	43.526111	5.071446	2023	[32]
Rapana venosa	Mediterranean Sea	43.540190	5.031618	2023	[32]
Rapana venosa	Mediterranean Sea	43.545856	5.030433	2023	[32]

SPECIES	AREA	LATITUDE	LONGITUDE	YEAR	REFERENCE
Rapana venosa	Mediterranean Sea	43.541641	5.014062	2023	[32]
Rapana venosa	Mediterranean Sea	43.523012	5.014930	2023	[32]
Rapana venosa	Mediterranean Sea	43.499586	4.999097	2023	[32]
Rapana venosa	Mediterranean Sea	43.475638	5.001782	2023	[32]
Rapana venosa	Mediterranean Sea	43.470824	5.033696	2023	[32]
Rapana venosa	Mediterranean Sea	43.448062	5.055507	2023	[32]
Rapana venosa	Mediterranean Sea	43.424991	5.061271	2023	[32]
Rapana venosa	Mediterranean Sea	43.401527	5.095174	2023	[32]
Rapana venosa	Mediterranean Sea	43.405386	5.115078	2023	[32]
Rapana venosa	Black Sea and adjacent waters	41.197200	37.043600	2011	[33]
Rapana venosa	Black Sea and adjacent waters	41.149600	37.287600	2011	[33]
Rapana venosa	Black Sea and adjacent waters	41.036300	37.506500	2011	[33]
Rapana venosa	Black Sea and adjacent waters	41.067700	37.780800	2011	[33]
Rapana venosa	Black Sea and adjacent waters	40.988300	37.899300	2011	[33]
Rapana venosa	Black Sea and adjacent waters	42.028300	35.125100	2010	[34]
Rapana venosa	Black Sea and adjacent waters	42.014800	35.146400	2010	[34]
Rapana venosa	Northwest Europe	42.487633	-8.855383	2003	[35]
Rapana venosa	Northwest Europe	43.468850	-8.217942	2023	[36]
Rapana venosa	Northwest Europe	42.613865	-8.808634	2023	[36]
Rapana venosa	Black Sea and adjacent waters	41.476100	36.136200	2021	[37]
Rapana venosa	Black Sea and adjacent waters	42.004500	35.197700	2020	[38]
Rapana venosa	Black Sea and adjacent waters	41.292100	36.361700	2020	[38]
Rapana venosa	Black Sea and adjacent waters	40.964000	39.915000	2020	[38]
Rapana venosa	Black Sea and adjacent waters	42.014800	35.146400	1997	[39]
Rapana venosa	Black Sea and adjacent waters	42.048500	35.046600	1997	[39]
Rapana venosa	Black Sea and adjacent waters	42.017100	35.173700	1997	[39]
Rapana venosa	Black Sea and adjacent waters	41.235702	29.137720	1969	[40]
Rapana venosa	Black Sea and adjacent waters	41.273041	31.369866	1969	[40]
Rapana venosa	Black Sea and adjacent waters	41.474170	31.765156	1969	[40]
Rapana venosa	Black Sea and adjacent waters	41.761421	32.362822	1970	[40]

SPECIES	AREA	LATITUDE	LONGITUDE	YEAR	REFERENCE
Rapana venosa	Black Sea and adjacent waters	42.016978	33.319847	1970	[40]
Rapana venosa	Black Sea and adjacent waters	41.951761	34.735343	1970	[40]
Rapana venosa	Black Sea and adjacent waters	41.962264	34.790973	1970	[40]
Rapana venosa	Black Sea and adjacent waters	41.808451	35.214347	1970	[40]
Rapana venosa	Black Sea and adjacent waters	41.640852	35.604823	1970	[40]
Rapana venosa	Black Sea and adjacent waters	41.303484	36.368326	1970	[40]
Rapana venosa	Black Sea and adjacent waters	41.874920	27.991160	1971	[40]
Rapana venosa	Black Sea and adjacent waters	41.349838	28.683819	1971	[40]
Rapana venosa	Black Sea and adjacent waters	42.034015	35.230150	1971	[40]
Rapana venosa	Black Sea and adjacent waters	41.630832	36.120503	1971	[40]
Rapana venosa	Black Sea and adjacent waters	41.273854	37.023190	1971	[40]
Rapana venosa	Black Sea and adjacent waters	41.092038	30.868093	1972	[40]
Rapana venosa	Black Sea and adjacent waters	41.209507	29.474731	1972	[40]
Rapana venosa	Black Sea and adjacent waters	45.779500	33.074400	2007	[41]
Rapana venosa	Mediterranean Sea	43.738900	13.233600	1987	[42]
Rapana venosa	Mediterranean Sea	43.568400	13.607700	1987	[42]
Rapana venosa	Mediterranean Sea	44.104700	12.597900	1989	[42]
Rapana venosa	Mediterranean Sea	44.069500	12.635700	1989	[42]
Rapana venosa	Mediterranean Sea	44.742200	12.354000	1990	[42]
Rapana venosa	Black Sea and adjacent waters	45.348590	32.974981	2020	[43]
Rapana venosa	Black Sea and adjacent waters	45.352754	32.998619	2020	[43]
Rapana venosa	Black Sea and adjacent waters	45.336969	32.978826	2020	[43]
Rapana venosa	Black Sea and adjacent waters	45.327634	32.986803	2020	[43]
Rapana venosa	Black Sea and adjacent waters	45.319975	33.010712	2020	[43]
Rapana venosa	Black Sea and adjacent waters	45.367912	33.031693	2020	[43]
Rapana venosa	Black Sea and adjacent waters	45.382015	33.058586	2020	[43]
Rapana venosa	Black Sea and adjacent waters	45.495200	36.021200	2006	[44]
Rapana venosa	Black Sea and adjacent waters	44.293410	28.844099	2012	[44]
Rapana venosa	Black Sea and adjacent waters	45.370918	31.689802	2012	[44]
Rapana venosa	Black Sea and adjacent waters	45.779571	33.074456	2012	[44]

SPECIES	AREA	LATITUDE	LONGITUDE	YEAR	REFERENCE
Rapana venosa	Black Sea and adjacent waters	45.169299	33.399471	2012	[44]
Rapana venosa	Black Sea and adjacent waters	44.621250	33.499180	2012	[44]
Rapana venosa	Black Sea and adjacent waters	44.410491	33.700150	2012	[44]
Rapana venosa	Black Sea and adjacent waters	44.928023	35.247884	2012	[44]
Rapana venosa	Black Sea and adjacent waters	45.244564	36.501356	2012	[44]
Rapana venosa	Black Sea and adjacent waters	45.350244	32.974673	2020	[45]
Rapana venosa	Black Sea and adjacent waters	45.333449	32.983720	2020	[45]
Rapana venosa	Black Sea and adjacent waters	45.353273	32.998860	2020	[45]
Rapana venosa	Black Sea and adjacent waters	45.319160	33.011318	2020	[45]
Rapana venosa	Black Sea and adjacent waters	45.369272	33.032160	2020	[45]
Rapana venosa	Black Sea and adjacent waters	45.384619	33.066665	2020	[45]
Rapana venosa	Black Sea and adjacent waters	45.313652	33.001900	2020	[45]
Rapana venosa	Black Sea and adjacent waters	45.328173	32.981259	2020	[45]
Rapana venosa	Black Sea and adjacent waters	45.339344	32.958796	2020	[45]
Rapana venosa	Black Sea and adjacent waters	45.351697	32.930658	2020	[45]
Rapana venosa	Northwest Europe	47.565898	-3.109898	1998	[46]
Rapana venosa	Northwest Europe	47.565898	-3.109898	1999	[46]
Rapana venosa	Northwest Europe	47.565898	-3.109898	2000	[46]
Rapana venosa	Mediterranean Sea	43.001944	13.870556	2021	[47]
Rapana venosa	Mediterranean Sea	43.088333	13.849167	2021	[47]
Rapana venosa	Black Sea and adjacent waters	43.776092	28.607639	2008	[48]
Rapana venosa	Northwest Europe	47.471417	-3.122071	1997	[49]
Rapana venosa	Mediterranean Sea	44.738144	12.386595	2004	[50]
Rapana venosa	Mediterranean Sea	44.265000	12.443333	2004	[50]
Rapana venosa	Mediterranean Sea	43.852905	13.026170	2004	[50]
		.	.		
Rapana venosa	Black Sea and adjacent waters	40.359167	27.853611	2011	[51]
Rapana venosa	Black Sea and adjacent waters	42.014800	35.146400	1995	[52]
Rapana venosa	Black Sea and adjacent waters	41.985000	35.109000	1995	[52]
Rapana venosa	Mediterranean Sea	45.411281	12.441374	1981	[53]

SPECIES	AREA	LATITUDE	LONGITUDE	YEAR	REFERENCE
Rapana venosa	Mediterranean Sea	44.473381	12.300229	1981	[53]
Rapana venosa	Mediterranean Sea	44.473381	12.300229	1982	[53]
Rapana venosa	Mediterranean Sea	45.316500	12.317700	1983	[53]
Rapana venosa	Mediterranean Sea	45.316500	12.317700	1983	[53]
Rapana venosa	Mediterranean Sea	44.497115	12.325378	1983	[53]
Rapana venosa	Mediterranean Sea	45.316500	12.317700	1983	[53]
Rapana venosa	Mediterranean Sea	44.810900	12.319400	1983	[53]
Rapana venosa	Mediterranean Sea	44.398500	12.591100	1983	[53]
Rapana venosa	Mediterranean Sea	44.556400	12.473600	1983	[53]
Rapana venosa	Mediterranean Sea	44.363700	12.324700	1983	[53]
Rapana venosa	Mediterranean Sea	44.495407	12.308323	1983	[53]
Rapana venosa	Mediterranean Sea	45.370300	12.405100	1984	[53]
Rapana venosa	Mediterranean Sea	45.217300	12.401000	1984	[53]
Rapana venosa	Mediterranean Sea	45.231100	12.312700	1984	[53]
Rapana venosa	Mediterranean Sea	45.434000	12.382000	1984	[53]
Rapana venosa	Mediterranean Sea	44.487900	12.448200	1984	[53]
Rapana venosa	Mediterranean Sea	44.497115	12.325378	1984	[53]
Rapana venosa	Mediterranean Sea	45.225000	12.282600	1984	[53]
Rapana venosa	Mediterranean Sea	44.558600	12.286800	1984	[53]
Rapana venosa	Mediterranean Sea	44.473381	12.300229	1984	[53]
Rapana venosa	Mediterranean Sea	44.473381	12.300229	1984	[53]
Rapana venosa	Mediterranean Sea	44.497115	12.325378	1984	[53]
Rapana venosa	Mediterranean Sea	45.225000	12.282600	1984	[53]
Rapana venosa	Mediterranean Sea	45.225000	12.282600	1984	[53]
Rapana venosa	Black Sea and adjacent waters	41.017100	39.722300	1999	[54]
Rapana venosa	Mediterranean Sea	44.738144	12.386595	1999	[54]
Rapana venosa	Mediterranean Sea	44.742200	12.354000	1999	[54]
Rapana venosa	Mediterranean Sea	44.203333	12.411667	1999	[54]
Rapana venosa	Mediterranean Sea	43.852905	13.026170	1999	[54]
Rapana venosa	Mediterranean Sea	44.738144	12.386595	2004	[54]

SPECIES	AREA	LATITUDE	LONGITUDE	YEAR	REFERENCE
Rapana venosa	Mediterranean Sea	44.203333	12.411667	2004	[54]
Rapana venosa	Mediterranean Sea	43.852905	13.026170	2004	[54]
Rapana venosa	Black Sea and adjacent waters	41.044460	40.518660	2005	[54]
Rapana venosa	Black Sea and adjacent waters	44.087227	39.061516	2006	[54]
Rapana venosa	Mediterranean Sea	42.177778	14.691667	2024	[55]
Rapana venosa	Black Sea and adjacent waters	43.000000	40.533333	1958	[56]
Rapana venosa	Mediterranean Sea	38.267911	15.636147	2007	[57]
Rapana venosa	Mediterranean Sea	40.942700	9.607300	2009	[58]
Rapana venosa	Mediterranean Sea	45.527300	13.566700	2008	[59]
Rapana venosa	Mediterranean Sea	39.241200	9.043300	2006	[60]
Rapana venosa	Mediterranean Sea	41.294400	13.019700	2006	[60]
Rapana venosa	Mediterranean Sea	38.267900	15.636100	2006	[60]
Rapana venosa	Mediterranean Sea	45.729641	13.684782	1983	[61]
Rapana venosa	Mediterranean Sea	45.729641	13.684782	1984	[61]
Rapana venosa	Black Sea and adjacent waters	42.033333	35.051667	2006	[62]
Rapana venosa	Black Sea and adjacent waters	42.034722	35.052222	2006	[62]
Rapana venosa	Black Sea and adjacent waters	42.034722	35.069167	2006	[62]
Rapana venosa	Black Sea and adjacent waters	42.034444	35.086667	2006	[62]
Rapana venosa	Black Sea and adjacent waters	42.034444	35.137500	2006	[62]
Rapana venosa	Black Sea and adjacent waters	42.000278	35.189722	2006	[62]
Rapana venosa	Black Sea and adjacent waters	42.000278	35.154722	2006	[62]
Rapana venosa	Black Sea and adjacent waters	42.000278	35.104722	2006	[62]
Rapana venosa	Black Sea and adjacent waters	41.965278	35.086944	2006	[62]
Rapana venosa	Black Sea and adjacent waters	41.916667	35.120833	2006	[62]
Rapana venosa	Black Sea and adjacent waters	41.866944	35.155278	2006	[62]
Rapana venosa	Black Sea and adjacent waters	41.783611	35.216667	2006	[62]
Rapana venosa	Black Sea and adjacent waters	41.909494	32.971397	2011	[63]
Rapana venosa	Black Sea and adjacent waters	41.979564	33.758519	2011	[63]
Rapana venosa	Black Sea and adjacent waters	41.952121	34.337867	2011	[63]
Rapana venosa	Black Sea and adjacent waters	41.951381	34.590917	2011	[63]

SPECIES	AREA	LATITUDE	LONGITUDE	YEAR	REFERENCE
Rapana venosa	Black Sea and adjacent waters	42.026566	34.908070	2011	[63]
Rapana venosa	Black Sea and adjacent waters	41.909494	32.971397	2012	[63]
Rapana venosa	Black Sea and adjacent waters	41.979564	33.758519	2012	[63]
Rapana venosa	Black Sea and adjacent waters	41.952121	34.337867	2012	[63]
Rapana venosa	Black Sea and adjacent waters	41.951381	34.590917	2012	[63]
Rapana venosa	Black Sea and adjacent waters	42.026566	34.908070	2012	[63]
Rapana venosa	Black Sea and adjacent waters	44.263000	28.628000	2013	[64]
Rapana venosa	Black Sea and adjacent waters	44.359000	28.712000	2013	[64]
Rapana venosa	Black Sea and adjacent waters	44.803000	29.425000	2013	[64]
Rapana venosa	Mediterranean Sea	45.501482	13.555711	1997	[65]
Rapana venosa	Black Sea and adjacent waters	40.972100	27.516400	1992	[66]
Rapana venosa	Black Sea and adjacent waters	40.959900	27.933700	2012	[67]
Rapana venosa	Northwest Europe	45.457100	-0.964200	2020	[68]
Rapana venosa	Northwest Europe	45.433800	-0.923500	2020	[68]
Rapana venosa	Northwest Europe	45.566700	-1.059200	2020	[68]
Rapana venosa	Northwest Europe	45.807100	-1.148900	2020	[68]
Rapana venosa	Northwest Europe	45.921300	-1.091000	2020	[68]
Rapana venosa	Northwest Europe	45.940800	-1.160500	2020	[68]
Rapana venosa	Northwest Europe	45.966100	-1.185500	2020	[68]
Rapana venosa	Northwest Europe	45.999200	-1.204800	2020	[68]
Rapana venosa	Northwest Europe	46.018400	-1.436700	2020	[68]
Rapana venosa	Northwest Europe	46.014400	-1.074900	2020	[68]
Rapana venosa	Northwest Europe	46.019100	-1.078000	2020	[68]
Rapana venosa	Northwest Europe	46.016400	-1.081600	2020	[68]
Rapana venosa	Northwest Europe	46.020600	-1.074900	2020	[68]
Rapana venosa	Northwest Europe	46.052100	-1.102500	2020	[68]
Rapana venosa	Northwest Europe	46.053100	-1.108400	2020	[68]
Rapana venosa	Northwest Europe	46.034000	-1.256300	2020	[68]
Rapana venosa	Northwest Europe	46.038200	-1.238400	2020	[68]
Rapana venosa	Northwest Europe	46.048700	-1.194500	2020	[68]

SPECIES	AREA	LATITUDE	LONGITUDE	YEAR	REFERENCE
Rapana venosa	Northwest Europe	46.049600	-1.186400	2020	[68]
Rapana venosa	Northwest Europe	46.075800	-1.161100	2020	[68]
Rapana venosa	Northwest Europe	46.081500	-1.163300	2020	[68]
Rapana venosa	Northwest Europe	46.087700	-1.165100	2020	[68]
Rapana venosa	Northwest Europe	46.093400	-1.163600	2020	[68]
Rapana venosa	Northwest Europe	46.100100	-1.162100	2020	[68]
Rapana venosa	Northwest Europe	46.105300	-1.148000	2020	[68]
Rapana venosa	Northwest Europe	46.125500	-1.152500	2020	[68]
Rapana venosa	Northwest Europe	46.121700	-1.180800	2020	[68]
Rapana venosa	Northwest Europe	46.122000	-1.185700	2020	[68]
Rapana venosa	Northwest Europe	46.121500	-1.190500	2020	[68]
Rapana venosa	Northwest Europe	46.126800	-1.173600	2020	[68]
Rapana venosa	Northwest Europe	46.126800	-1.179500	2020	[68]
Rapana venosa	Northwest Europe	46.136300	-1.158500	2020	[68]
Rapana venosa	Northwest Europe	46.134300	-1.167400	2020	[68]
Rapana venosa	Northwest Europe	46.195000	-1.256700	2020	[68]
Rapana venosa	Northwest Europe	46.194600	-1.273100	2020	[68]
Rapana venosa	Northwest Europe	46.202600	-1.275100	2020	[68]
Rapana venosa	Northwest Europe	45.459400	-0.969400	2021	[68]
Rapana venosa	Northwest Europe	45.488900	-1.013400	2021	[68]
Rapana venosa	Northwest Europe	45.218900	-1.229000	2021	[68]
Rapana venosa	Black Sea and adjacent waters	42.016900	35.211200	1999	[69]
Rapana venosa	Mediterranean Sea	41.143624	9.494478	1995	[70]
Rapana venosa	Mediterranean Sea	40.914827	9.569928	1998	[70]
Rapana venosa	Mediterranean Sea	40.914827	9.569928	2001	[70]
Rapana venosa	Mediterranean Sea	43.474996	5.005145	2020	[71]
Rapana venosa	Mediterranean Sea	43.474996	5.005145	2020	[71]
Rapana venosa	Northwest Europe	46.145024	-1.184730	2020	[71]
Rapana venosa	Mediterranean Sea	43.474996	5.005145	2021	[71]
Rapana venosa	Mediterranean Sea	43.405305	5.110101	2021	[71]

SPECIES	AREA	LATITUDE	LONGITUDE	YEAR	REFERENCE
Rapana venosa	Black Sea and adjacent waters	40.915000	40.116000	1992	[72]
Rapana venosa	Black Sea and adjacent waters	40.938000	40.058000	1992	[72]
Rapana venosa	Black Sea and adjacent waters	40.950000	39.833000	1992	[72]
Rapana venosa	Black Sea and adjacent waters	40.995000	39.789000	1992	[72]
Rapana venosa	Black Sea and adjacent waters	40.980000	39.850000	1992	[72]
Rapana venosa	Black Sea and adjacent waters	40.890000	40.200000	1992	[72]
Rapana venosa	Black Sea and adjacent waters	41.083333	39.500000	1991	[73]
Rapana venosa	Black Sea and adjacent waters	40.933333	40.200000	1991	[73]
Rapana venosa	Black Sea and adjacent waters	40.958900	39.867700	1991	[73]
Rapana venosa	Black Sea and adjacent waters	40.941700	40.057400	1991	[73]
Rapana venosa	Black Sea and adjacent waters	40.918500	40.110500	1991	[73]
Rapana venosa	Black Sea and adjacent waters	40.913400	40.119900	1991	[73]
Rapana venosa	Black Sea and adjacent waters	40.998900	39.788500	1991	[73]
Rapana venosa	Black Sea and adjacent waters	41.058100	39.530700	1991	[73]
Rapana venosa	Black Sea and adjacent waters	41.002354	39.776086	1991	[73]
Rapana venosa	Black Sea and adjacent waters	41.012022	39.707285	1991	[73]
Rapana venosa	Black Sea and adjacent waters	41.083333	39.500000	1991	[73]
Rapana venosa	Mediterranean Sea	36.869600	27.571200	1995	[74]
Rapana venosa	Black Sea and adjacent waters	41.103200	39.434200	2018	[75]
Rapana venosa	Black Sea and adjacent waters	41.095300	39.392200	2018	[75]
Rapana venosa	Black Sea and adjacent waters	41.052300	39.277500	2018	[75]
Rapana venosa	Black Sea and adjacent waters	41.092600	39.483000	2018	[75]
Rapana venosa	Black Sea and adjacent waters	41.091000	39.365700	2018	[75]
Rapana venosa	Black Sea and adjacent waters	41.065800	39.326600	2018	[75]
Rapana venosa	Black Sea and adjacent waters	40.954059	40.254592	2015	[76]
Rapana venosa	Black Sea and adjacent waters	41.171261	40.797308	2015	[76]
Rapana venosa	Mediterranean Sea	41.879500	15.746800	2006	[77]
Rapana venosa	Mediterranean Sea	41.879500	15.746800	2007	[77]
Rapana venosa	Black Sea and adjacent waters	42.266200	27.769100	2020	[78]
Rapana venosa	Mediterranean Sea	44.501342	12.616253	1973	[79]

SPECIES	AREA	LATITUDE	LONGITUDE	YEAR	REFERENCE
Rapana venosa	Mediterranean Sea	38.267911	15.636147	2008	[80]
Rapana venosa	Black Sea and adjacent waters	42.502900	27.486800	2003	[81]
Rapana venosa	Mediterranean Sea	43.310900	13.741300	2003	[81]
Rapana venosa	Mediterranean Sea	40.469200	17.231200	2003	[81]
Rapana venosa	Mediterranean Sea	40.049900	17.989200	2003	[81]
Rapana venosa	Mediterranean Sea	44.477600	12.319200	2003	[81]
Rapana venosa	Black Sea and adjacent waters	44.486300	34.164500	1954	[82]
Rapana venosa	Black Sea and adjacent waters	44.621250	33.499180	1954	[82]
Rapana venosa	Black Sea and adjacent waters	44.263000	28.628600	1966	[83]
Rapana venosa	Black Sea and adjacent waters	44.071800	28.645400	1966	[83]
Rapana venosa	Black Sea and adjacent waters	44.263000	28.628600	1970	[83]
Rapana venosa	Black Sea and adjacent waters	44.428600	28.771700	1970	[83]
Rapana venosa	Black Sea and adjacent waters	44.638500	29.160300	1970	[83]
Rapana venosa	Black Sea and adjacent waters	44.071800	28.645400	1972	[83]
Rapana venosa	Black Sea and adjacent waters	44.338900	28.701900	1972	[83]
Rapana venosa	Black Sea and adjacent waters	42.698200	27.899900	1972	[83]
Rapana venosa	Black Sea and adjacent waters	44.338900	28.701900	1972	[83]
Rapana venosa	Black Sea and adjacent waters	42.426800	27.709500	1972	[83]
Rapana venosa	Black Sea and adjacent waters	43.813500	28.592400	1972	[83]
Rapana venosa	Black Sea and adjacent waters	46.440760	30.772241	1974	[84]
Rapana venosa	Black Sea and adjacent waters	41.691111	35.438056	2015	[85]
Rapana venosa	Black Sea and adjacent waters	41.686111	35.420556	2015	[85]
Rapana venosa	Black Sea and adjacent waters	41.680192	35.661944	2015	[85]
Rapana venosa	Black Sea and adjacent waters	41.673247	35.669722	2015	[85]
Rapana venosa	Black Sea and adjacent waters	42.099262	35.020000	2015	[85]
Rapana venosa	Black Sea and adjacent waters	42.107337	35.034167	2015	[85]
Rapana venosa	Black Sea and adjacent waters	42.031408	35.141944	2015	[85]
Rapana venosa	Black Sea and adjacent waters	42.043649	35.139444	2015	[85]
Rapana venosa	Mediterranean Sea	43.454782	5.106124	2015	[86]
Rapana venosa	Black Sea and adjacent waters	45.254000	30.195600	2004	[87]

SPECIES	AREA	LATITUD E	LONGITUD E	YEA R	REFERENCE
Rapana venosa	Black Sea and adjacent waters	46.375097	30.752439	2004	[87]
Rapana venosa	Black Sea and adjacent waters	46.340400	31.659300	2004	[87]
Rapana venosa	Black Sea and adjacent waters	45.779500	33.074400	2004	[87]
Rapana venosa	Black Sea and adjacent waters	46.440760	30.772241	2004	[88]
Rapana venosa	Black Sea and adjacent waters	45.248465	30.203890	2004	[898]
Rapana venosa	Black Sea and adjacent waters	44.837758	34.975699	2004	[88]
Rapana venosa	Black Sea and adjacent waters	45.359400	29.804500	2005	[88]
Rapana venosa	Black Sea and adjacent waters	46.340400	31.659300	2005	[88]
Rapana venosa	Black Sea and adjacent waters	46.440760	30.772241	2006	[88]
Rapana venosa	Black Sea and adjacent waters	45.779571	33.074456	2008	[88]
Rapana venosa	Black Sea and adjacent waters	45.311300	32.659500	2009	[88]
Rapana venosa	Black Sea and adjacent waters	45.779571	33.074456	2011	[88]
Rapana venosa	Black Sea and adjacent waters	44.638500	29.160300	2005	[89]
Rapana venosa	Black Sea and adjacent waters	44.713000	29.343900	1961	[90]
Rapana venosa	Black Sea and adjacent waters	44.713000	29.343900	1964	[90]
Rapana venosa	Black Sea and adjacent waters	45.345700	36.485200	1948	[91]
Rapana venosa	Black Sea and adjacent waters	42.578212	27.494539	2015	[92]
Rapana venosa	Black Sea and adjacent waters	42.550076	27.478017	2015	[92]
Rapana venosa	Black Sea and adjacent waters	45.182238	29.807994	2018	[93]
Rapana venosa	Black Sea and adjacent waters	46.227284	31.618139	2018	[93]
Rapana venosa	Black Sea and adjacent waters	45.337100	29.863800	2018	[94]
Rapana venosa	Black Sea and adjacent waters	45.561500	29.726900	2018	[94]
Rapana venosa	Black Sea and adjacent waters	45.522400	29.936300	2018	[94]
Rapana venosa	Black Sea and adjacent waters	45.403900	30.301600	2018	[94]
Rapana venosa	Black Sea and adjacent waters	45.656900	29.905000	2018	[94]
Rapana venosa	Black Sea and adjacent waters	45.725600	30.007100	2018	[94]
Rapana venosa	Black Sea and adjacent waters	45.624900	30.107700	2018	[94]
Rapana venosa	Black Sea and adjacent waters	45.801800	30.139900	2018	[94]
Rapana venosa	Black Sea and adjacent waters	45.778600	30.172700	2018	[94]
Rapana venosa	Black Sea and adjacent waters	45.724800	30.233900	2018	[94]

SPECIES	AREA	LATITUDE	LONGITUDE	YEAR	REFERENCE
Rapana venosa	Black Sea and adjacent waters	45.663000	30.295200	2018	[94]
Rapana venosa	Black Sea and adjacent waters	45.518700	30.136700	2018	[94]
Rapana venosa	Black Sea and adjacent waters	45.872300	30.269800	2018	[94]
Rapana venosa	Black Sea and adjacent waters	45.983200	30.369500	2018	[94]
Rapana venosa	Black Sea and adjacent waters	45.860600	30.437900	2018	[94]
Rapana venosa	Black Sea and adjacent waters	46.100900	30.508900	2018	[94]
Rapana venosa	Black Sea and adjacent waters	46.199600	30.617300	2018	[94]
Rapana venosa	Black Sea and adjacent waters	46.283200	30.711200	2018	[94]
Rapana venosa	Black Sea and adjacent waters	45.724800	30.508900	2018	[94]
Rapana venosa	Black Sea and adjacent waters	45.936100	30.591000	2018	[94]
Rapana venosa	Black Sea and adjacent waters	46.143200	30.732100	2018	[94]
Rapana venosa	Black Sea and adjacent waters	45.964200	30.732100	2018	[94]
Rapana venosa	Black Sea and adjacent waters	45.826700	30.673400	2018	[94]
Rapana venosa	Black Sea and adjacent waters	45.572500	30.627400	2018	[94]
Rapana venosa	Black Sea and adjacent waters	45.724800	30.822800	2018	[94]
Rapana venosa	Black Sea and adjacent waters	46.424200	30.861200	2018	[94]
Rapana venosa	Black Sea and adjacent waters	46.333900	31.000100	2018	[94]
Rapana venosa	Black Sea and adjacent waters	46.155400	30.987700	2018	[94]
Rapana venosa	Black Sea and adjacent waters	46.035800	30.904300	2018	[94]
Rapana venosa	Black Sea and adjacent waters	45.923100	30.904300	2018	[94]
Rapana venosa	Black Sea and adjacent waters	46.506000	31.064000	2018	[94]
Rapana venosa	Black Sea and adjacent waters	46.602600	31.179600	2018	[94]
Rapana venosa	Black Sea and adjacent waters	46.547400	31.163600	2018	[94]
Rapana venosa	Black Sea and adjacent waters	46.506000	31.281100	2018	[94]
Rapana venosa	Black Sea and adjacent waters	46.456400	31.109600	2018	[94]
Rapana venosa	Black Sea and adjacent waters	46.385200	31.083200	2018	[94]
Rapana venosa	Black Sea and adjacent waters	46.237900	31.087900	2018	[94]
Rapana venosa	Black Sea and adjacent waters	46.022400	31.113600	2018	[94]
Rapana venosa	Black Sea and adjacent waters	46.459400	31.357900	2018	[94]
Rapana venosa	Black Sea and adjacent waters	46.486100	31.447000	2018	[94]

SPECIES	AREA	LATITUDE	LONGITUDE	YEAR	REFERENCE
Rapana venosa	Black Sea and adjacent waters	46.382100	31.281100	2018	[94]
Rapana venosa	Black Sea and adjacent waters	46.210800	31.302800	2018	[94]
Rapana venosa	Black Sea and adjacent waters	46.276200	31.498300	2018	[94]
Rapana venosa	Black Sea and adjacent waters	45.891400	31.254900	2018	[94]
Rapana venosa	Black Sea and adjacent waters	46.200400	31.438000	2018	[94]
Rapana venosa	Black Sea and adjacent waters	46.167100	31.537900	2018	[94]
Rapana venosa	Black Sea and adjacent waters	46.160000	31.741300	2018	[94]
Rapana venosa	Black Sea and adjacent waters	46.092200	31.881000	2018	[94]
Rapana venosa	Black Sea and adjacent waters	46.072000	31.338400	2018	[94]
Rapana venosa	Black Sea and adjacent waters	45.983500	31.303600	2018	[94]
Rapana venosa	Black Sea and adjacent waters	46.021200	31.656100	2018	[94]
Rapana venosa	Black Sea and adjacent waters	46.019200	31.789500	2018	[94]
Rapana venosa	Mediterranean Sea	43.316000	13.728400	2001	[95]
Rapana venosa	Northwest Europe	46.009415	-1.264743	2011	[96]
Rapana venosa	Northwest Europe	47.551983	-2.921283	2003	[97]
Rapana venosa	Northwest Europe	47.471417	-3.122071	2003	[97]
Rapana venosa	Northwest Europe	47.551983	-2.921283	2004	[98]
Rapana venosa	Northwest Europe	47.471417	-3.122071	2005	[99]
Rapana venosa	Northwest Europe	47.471417	-3.122071	2006	[100]
Rapana venosa	Northwest Europe	48.523611	-4.770833	2009	[101]
Rapana venosa	Northwest Europe	47.568494	-3.103375	2009	[101]
Rapana venosa	Northwest Europe	47.583392	-2.994217	2009	[101]
Rapana venosa	Northwest Europe	47.574600	-2.999000	2012	[102]
Rapana venosa	Black Sea and adjacent waters	44.808999	29.322686	1968	[103]
Rapana venosa	Black Sea and adjacent waters	44.728300	29.054300	1968	[103]
Rapana venosa	Black Sea and adjacent waters	44.215657	28.645598	1968	[103]
Rapana venosa	Mediterranean Sea	41.038680	17.141920	2006	[104]
Rapana venosa	Mediterranean Sea	44.786040	12.360250	2012	[104]
Rapana venosa	Mediterranean Sea	44.209520	12.396520	2017	[104]
Rapana venosa	Mediterranean Sea	44.221370	12.386550	2018	[104]

SPECIES	AREA	LATITUDE	LONGITUDE	YEAR	REFERENCE
Rapana venosa	Mediterranean Sea	42.217140	14.596130	2019	[104]
Rapana venosa	Black Sea and adjacent waters	40.870760	29.074700	2020	[104]
Rapana venosa	Black Sea and adjacent waters	42.015580	35.192150	2020	[104]
Rapana venosa	Mediterranean Sea	44.801960	12.274830	2020	[104]
Rapana venosa	Mediterranean Sea	44.301180	12.348120	2020	[104]
Rapana venosa	Black Sea and adjacent waters	41.013000	39.614190	2021	[104]
Rapana venosa	Mediterranean Sea	42.675250	14.016010	2021	[104]
Rapana venosa	Mediterranean Sea	43.478570	5.001720	2021	[104]
Rapana venosa	Mediterranean Sea	43.405540	5.114730	2021	[104]
Rapana venosa	Mediterranean Sea	44.793490	12.400870	2021	[104]
Rapana venosa	Mediterranean Sea	43.474770	5.001240	2021	[104]
Rapana venosa	Mediterranean Sea	44.499090	12.307160	2021	[104]
Rapana venosa	Mediterranean Sea	43.497190	5.067060	2021	[104]
Rapana venosa	Mediterranean Sea	44.200840	12.405200	2021	[104]
Rapana venosa	Mediterranean Sea	44.207510	12.398860	2021	[104]
Rapana venosa	Mediterranean Sea	44.251660	12.364970	2021	[104]
Rapana venosa	Black Sea and adjacent waters	44.971470	37.266260	2022	[104]
Rapana venosa	Mediterranean Sea	43.404920	5.114710	2022	[104]
Rapana venosa	Mediterranean Sea	43.404940	5.114900	2022	[104]
Rapana venosa	Mediterranean Sea	44.497520	12.288430	2022	[104]
Rapana venosa	Mediterranean Sea	44.554480	12.285190	2022	[104]
Rapana venosa	Mediterranean Sea	43.405250	5.115190	2022	[104]
Rapana venosa	Black Sea and adjacent waters	42.339070	27.730730	2023	[104]
Rapana venosa	Black Sea and adjacent waters	42.252400	27.751820	2023	[104]
Rapana venosa	Black Sea and adjacent waters	42.252370	27.751760	2023	[104]
Rapana venosa	Black Sea and adjacent waters	41.313540	36.578860	2023	[104]
Rapana venosa	Black Sea and adjacent waters	41.311410	36.576790	2023	[104]
Rapana venosa	Mediterranean Sea	43.404640	5.113170	2023	[104]
Rapana venosa	Mediterranean Sea	43.496160	4.999970	2023	[104]
Rapana venosa	Mediterranean Sea	43.477660	5.001910	2023	[104]

SPECIES	AREA	LATITUDE	LONGITUDE	YEAR	REFERENCE
Rapana venosa	Black Sea and adjacent waters	41.246040	29.011930	2024	[104]
Rapana venosa	Black Sea and adjacent waters	40.473530	27.487490	2024	[104]
Rapana venosa	Mediterranean Sea	44.388310	12.321430	2024	[104]
Rapana venosa	Mediterranean Sea	43.963310	12.738430	2024	[104]
Rapana venosa	Mediterranean Sea	43.438820	5.195090	2024	[104]
Rapana venosa	Mediterranean Sea	43.405750	5.116430	2024	[104]
Rapana venosa	Mediterranean Sea	43.470600	5.020060	2024	[104]
Rapana venosa	Mediterranean Sea	43.545490	5.030880	2024	[104]
Rapana venosa	Black Sea and adjacent waters	41.293180	36.339670	2025	[104]
Rapana venosa	Black Sea and adjacent waters	40.951720	29.095030	2025	[104]
Rapana venosa	Black Sea and adjacent waters	41.195560	29.118420	2025	[104]
Rapana venosa	Black Sea and adjacent waters	41.223550	29.110810	2025	[104]
Rapana venosa	Black Sea and adjacent waters	40.885390	29.055210	2025	[104]
Rapana venosa	Mediterranean Sea	42.263680	18.857180	2025	[104]
Rapana venosa	Mediterranean Sea	43.401300	5.134560	2025	[104]
Rapana venosa	Northwest Europe	43.656000	-1.438190	2025	[104]
Rapana venosa	Black Sea and adjacent waters	43.090200	40.619100	1960	[105]
Rapana venosa	Black Sea and adjacent waters	43.086200	40.687500	1960	[105]
Rapana venosa	Black Sea and adjacent waters	45.096800	36.902000	1960	[105]
Rapana venosa	Black Sea and adjacent waters	45.273300	36.439300	1960	[105]
Rapana venosa	Black Sea and adjacent waters	45.318200	36.533600	1960	[105]
Rapana venosa	Black Sea and adjacent waters	44.147431	38.974473	1960	[105]
Rapana venosa	Black Sea and adjacent waters	44.113877	38.996504	1960	[105]
Rapana venosa	Black Sea and adjacent waters	45.055883	36.912068	1960	[105]
Rapana venosa	Black Sea and adjacent waters	45.109706	36.838583	1960	[105]
Rapana venosa	Black Sea and adjacent waters	45.068038	36.820188	1960	[105]
Rapana venosa	Black Sea and adjacent waters	45.100006	36.738238	1960	[105]
Rapana venosa	Black Sea and adjacent waters	45.070355	36.705433	1960	[105]
Rapana venosa	Black Sea and adjacent waters	45.123369	36.534559	1960	[105]
Rapana venosa	Black Sea and adjacent waters	45.108909	36.652225	1960	[105]

SPECIES	AREA	LATITUDE	LONGITUDE	YEAR	REFERENCE
Rapana venosa	Black Sea and adjacent waters	45.131604	36.584487	1960	[105]
Rapana venosa	Black Sea and adjacent waters	45.202130	36.501811	1960	[105]
Rapana venosa	Black Sea and adjacent waters	45.200624	36.555145	1960	[105]
Rapana venosa	Black Sea and adjacent waters	45.244564	36.501356	1960	[105]
Rapana venosa	Black Sea and adjacent waters	45.236351	36.422601	1960	[105]
Rapana venosa	Black Sea and adjacent waters	45.166032	36.417651	1960	[105]
Rapana venosa	Black Sea and adjacent waters	45.101603	36.458920	1960	[105]
Rapana venosa	Black Sea and adjacent waters	45.048733	36.057590	1960	[105]
Rapana venosa	Black Sea and adjacent waters	45.015324	36.942123	1960	[105]
Rapana venosa	Black Sea and adjacent waters	45.036906	36.782133	1960	[105]
Rapana venosa	Black Sea and adjacent waters	45.039513	36.576642	1960	[105]
Rapana venosa	Black Sea and adjacent waters	45.194000	36.591900	1958	[106]
Rapana venosa	Black Sea and adjacent waters	45.194000	36.591900	1989	[107]
Rapana venosa	Black Sea and adjacent waters	43.178350	27.932300	2015	[108]
Rapana venosa	Black Sea and adjacent waters	43.103500	27.921667	2015	[108]
Rapana venosa	Black Sea and adjacent waters	43.103333	27.937833	2015	[108]
Rapana venosa	Black Sea and adjacent waters	43.103167	27.951833	2015	[108]
Rapana venosa	Black Sea and adjacent waters	47.560536	0.000000	2001	[109]
Rapana venosa	Northwest Europe	47.007155	-2.072627	2002	[109]
Rapana venosa	Black Sea and adjacent waters	43.172894	27.946569	1956	[110]
Rapana venosa	Black Sea and adjacent waters	40.964444	39.875000	2010	[111]
Rapana venosa	Black Sea and adjacent waters	40.930000	40.171389	2010	[111]
Rapana venosa	Mediterranean Sea	43.188000	13.797700	1988	[112]
Rapana venosa	Mediterranean Sea	44.265500	12.360200	1990	[112]
Rapana venosa	Black Sea and adjacent waters	41.397332	36.211438	2014	[113]
Rapana venosa	Black Sea and adjacent waters	41.259300	36.494500	2014	[113]
Rapana venosa	Northwest Europe	52.166667	3.916667	2005	[114]
Rapana venosa	Northwest Europe	51.650000	2.116667	2005	[114]
Rapana venosa	Black Sea and adjacent waters	44.753500	37.375300	2021	[115]
Rapana venosa	Black Sea and adjacent waters	41.132208	30.608399	1975	[116]

SPECIES	AREA	LATITUDE	LONGITUDE	YEAR	REFERENCE
Rapana venosa	Black Sea and adjacent waters	41.073436	31.014413	1977	[116]
Rapana venosa	Black Sea and adjacent waters	40.293278	26.525634	1984	[116]
Rapana venosa	Black Sea and adjacent waters	40.393879	28.795309	1984	[116]
Rapana venosa	Black Sea and adjacent waters	40.953922	39.901228	1986	[116]
Rapana venosa	Black Sea and adjacent waters	44.959100	35.259200	2001	[117]
Rapana venosa	Black Sea and adjacent waters	44.914500	35.231500	2001	[117]
Rapana venosa	Black Sea and adjacent waters	44.955240	35.267600	2001	[117]
Rapana venosa	Black Sea and adjacent waters	44.912500	35.218300	2001	[117]
Rapana venosa	Black Sea and adjacent waters	44.910000	35.205000	2001	[117]
Rapana venosa	Black Sea and adjacent waters	45.306900	36.621700	2003	[118]
Rapana venosa	Black Sea and adjacent waters	43.273800	28.230500	1984	[119]
Rapana venosa	Black Sea and adjacent waters	45.495200	36.021200	1956	[120]
Rapana venosa	Black Sea and adjacent waters	44.580700	33.370700	2009	[121]
Rapana venosa	Black Sea and adjacent waters	44.621250	33.499180	2009	[121]
Rapana venosa	Black Sea and adjacent waters	45.195200	36.597700	2009	[121]
Rapana venosa	Black Sea and adjacent waters	44.883500	37.293900	2009	[121]
Rapana venosa	Black Sea and adjacent waters	44.573400	37.981130	2009	[121]
Rapana venosa	Black Sea and adjacent waters	44.249950	38.817350	2009	[121]
Rapana venosa	Black Sea and adjacent waters	43.588530	39.706930	2009	[121]
Rapana venosa	Black Sea and adjacent waters	44.575700	37.979100	2010	[121]
Rapana venosa	Black Sea and adjacent waters	45.311300	32.659500	2011	[121]
Rapana venosa	Black Sea and adjacent waters	45.328100	32.981200	2011	[121]
Rapana venosa	Black Sea and adjacent waters	44.938072	37.300938	2010	[122]
Rapana venosa	Black Sea and adjacent waters	44.954008	37.285477	2010	[122]
Rapana venosa	Mediterranean Sea	42.438269	18.517550	2017	[123]
Rapana venosa	Mediterranean Sea	40.519524	22.936767	1986	[124]
Rapana venosa	Mediterranean Sea	40.597941	22.861688	1988	[124]
Rapana venosa	Black Sea and adjacent waters	45.254000	30.195600	2008	[125]
Rapana venosa	Black Sea and adjacent waters	46.465700	30.766200	2008	[125]
Rapana venosa	Black Sea and adjacent waters	45.311300	32.659500	2009	[125]

SPECIES	AREA	LATITUDE	LONGITUDE	YEAR	REFERENCE
Rapana venosa	Black Sea and adjacent waters	41.015756	38.843342	2014	[126]
Rapana venosa	Black Sea and adjacent waters	40.987857	37.929905	2015	[126]
Rapana venosa	Black Sea and adjacent waters	45.250000	30.200000	2010	[127]
Rapana venosa	Black Sea and adjacent waters	45.166667	30.083333	2010	[127]
Rapana venosa	Black Sea and adjacent waters	45.333333	30.300000	2010	[127]
Rapana venosa	Mediterranean Sea	44.810900	12.319400	1982	[128]
Rapana venosa	Mediterranean Sea	43.454782	5.106124	2015	[129]
Rapana venosa	Northwest Europe	47.574600	-2.999000	2012	[130]
Rapana venosa	Northwest Europe	46.145024	-1.184730	2020	[131]
Rapana venosa	Black Sea and adjacent waters	44.928000	35.247000	2008	[132]
Rapana venosa	Black Sea and adjacent waters	44.948000	35.247000	2008	[132]
Rapana venosa	Black Sea and adjacent waters	44.410400	33.700100	2008	[132]
Rapana venosa	Mediterranean Sea	45.413530	12.433465	1975	[133]
Rapana venosa	Mediterranean Sea	45.323373	12.383739	1975	[133]
Rapana venosa	Black Sea and adjacent waters	44.071800	28.645400	2008	[134]
Rapana venosa	Mediterranean Sea	32.407300	34.864600	2002	[135]
Rapana venosa	Black Sea and adjacent waters	45.194000	36.591900	2004	[136]
Rapana venosa	Black Sea and adjacent waters	44.883500	37.293900	2004	[136]
Rapana venosa	Black Sea and adjacent waters	44.415400	38.202300	2004	[136]
Rapana venosa	Black Sea and adjacent waters	44.185800	38.883500	2004	[136]
Rapana venosa	Black Sea and adjacent waters	43.666300	39.606300	2004	[136]
Rapana venosa	Black Sea and adjacent waters	43.086200	40.687500	2004	[136]
		.	.		
Rapana venosa	Black Sea and adjacent waters	46.440760	30.772241	2015	[137]
Rapana venosa	Black Sea and adjacent waters	42.016100	35.168800	2005	[138]
Rapana venosa	Black Sea and adjacent waters	43.189200	27.977900	2006	[139]
Rapana venosa	Black Sea and adjacent waters	43.204800	27.871200	2006	[139]
Rapana venosa	Mediterranean Sea	42.458700	14.238300	1982	[140]
Rapana venosa	Mediterranean Sea	42.416400	14.302200	1995	[140]
Rapana venosa	Mediterranean Sea	42.381500	14.369100	1995	[140]

SPECIES	AREA	LATITUDE	LONGITUDE	YEAR	REFERENCE
Rapana venosa	Mediterranean Sea	42.344000	14.415900	1996	[140]
Rapana venosa	Mediterranean Sea	42.509500	14.172800	1999	[140]
Rapana venosa	Northwest Europe	52.200000	4.016667	2005	[141]
Rapana venosa	Black Sea and adjacent waters	41.429330	41.419060	2021	[142]
Rapana venosa	Black Sea and adjacent waters	41.044460	40.518660	2021	[142]
Rapana venosa	Black Sea and adjacent waters	41.017100	39.722300	2021	[142]
Rapana venosa	Black Sea and adjacent waters	40.917470	38.430170	2021	[142]
Rapana venosa	Black Sea and adjacent waters	41.109420	37.648220	2021	[142]
Rapana venosa	Black Sea and adjacent waters	41.630830	36.120500	2021	[142]
Rapana venosa	Black Sea and adjacent waters	42.034010	35.230150	2021	[142]
Rapana venosa	Black Sea and adjacent waters	42.016970	33.319840	2021	[142]
Rapana venosa	Black Sea and adjacent waters	40.668100	28.112200	1990	[143]
Rapana venosa	Mediterranean Sea	43.429600	10.390800	1987	[144]
Rapana venosa	Northwest Europe	46.145024	-1.184730	2019	[145]
Rapana venosa	Northwest Europe	46.238370	-1.376579	2019	[145]
Rapana venosa	Black Sea and adjacent waters	44.486300	34.164500	1970	[146]
Rapana venosa	Black Sea and adjacent waters	44.837700	34.975600	1981	[146]
Rapana venosa	Black Sea and adjacent waters	45.194000	36.591900	1981	[146]
Rapana venosa	Black Sea and adjacent waters	44.753500	37.375300	1981	[146]
Rapana venosa	Black Sea and adjacent waters	43.893900	39.326300	1987	[146]
Rapana venosa	Black Sea and adjacent waters	45.337800	33.007600	2007	[146]
Rapana venosa	Black Sea and adjacent waters	45.221500	36.609250	2007	[146]
Rapana venosa	Black Sea and adjacent waters	45.068700	36.991200	2007	[146]
Rapana venosa	Northwest Europe	52.450300	4.553200	2012	[147]
Rapana venosa	Black Sea and adjacent waters	43.411700	28.338100	2020	[148]
Rapana venosa	Black Sea and adjacent waters	43.403500	28.157200	2020	[148]
Rapana venosa	Black Sea and adjacent waters	43.189200	27.977900	2020	[148]
Rapana venosa	Black Sea and adjacent waters	42.562100	27.615600	2020	[148]
Rapana venosa	Black Sea and adjacent waters	43.632600	27.657000	2020	[148]
Rapana venosa	Black Sea and adjacent waters	42.658600	27.750400	2021	[148]

SPECIES	AREA	LATITUDE	LONGITUDE	YEAR	REFERENCE
Rapana venosa	Black Sea and adjacent waters	42.426800	27.709500	2021	[148]
Rapana venosa	Black Sea and adjacent waters	43.367300	28.088900	2015	[149]
Rapana venosa	Black Sea and adjacent waters	42.426800	27.709500	2015	[149]
Rapana venosa	Black Sea and adjacent waters	43.411700	28.338100	2019	[150]
Rapana venosa	Black Sea and adjacent waters	43.189200	27.977900	2019	[150]
Rapana venosa	Black Sea and adjacent waters	43.318600	28.464700	2017	[151]
Rapana venosa	Black Sea and adjacent waters	42.337600	27.847400	2017	[151]
Rapana venosa	Black Sea and adjacent waters	44.620389	33.503778	2020	[152]
Rapana venosa	Mediterranean Sea	43.829100	13.057600	2007	[153]
Rapana venosa	Mediterranean Sea	43.848100	13.026300	2018	[153]
Rapana venosa	Mediterranean Sea	43.855200	13.037100	2018	[153]
Rapana venosa	Black Sea and adjacent waters	44.728300	29.054300	1968	[154]
Rapana venosa	Black Sea and adjacent waters	43.176390	27.958830	2016	[155]
Rapana venosa	Black Sea and adjacent waters	43.033500	27.889800	1995	[156]
Rapana venosa	Black Sea and adjacent waters	42.960600	27.898900	1995	[156]
Rapana venosa	Black Sea and adjacent waters	43.274900	28.055900	1995	[156]
Rapana venosa	Black Sea and adjacent waters	43.591000	28.465600	1995	[156]
Rapana venosa	Black Sea and adjacent waters	44.838800	29.660800	2009	[157]
Rapana venosa	Black Sea and adjacent waters	44.829300	29.651500	2010	[157]
Rapana venosa	Black Sea and adjacent waters	44.621000	29.130900	2010	[157]
Rapana venosa	Black Sea and adjacent waters	44.828200	29.651200	2010	[157]
Rapana venosa	Black Sea and adjacent waters	44.828900	29.650200	2011	[157]
Rapana venosa	Black Sea and adjacent waters	44.621500	29.103000	2011	[157]
Rapana venosa	Black Sea and adjacent waters	44.827600	29.650200	2012	[157]
Rapana venosa	Black Sea and adjacent waters	44.654300	29.021000	2012	[157]
Rapana venosa	Black Sea and adjacent waters	44.619400	29.105000	2012	[157]
Rapana venosa	Black Sea and adjacent waters	44.048700	28.659100	2014	[157]
Rapana venosa	Black Sea and adjacent waters	44.020200	28.674400	2014	[157]
Rapana venosa	Black Sea and adjacent waters	44.005500	28.681000	2014	[157]
Rapana venosa	Black Sea and adjacent waters	43.982100	28.677600	2014	[157]

SPECIES	AREA	LATITUDE	LONGITUDE	YEAR	REFERENCE
Rapana venosa	Black Sea and adjacent waters	43.960500	28.667400	2014	[157]
Rapana venosa	Black Sea and adjacent waters	43.956100	28.648600	2014	[157]
Rapana venosa	Black Sea and adjacent waters	43.943800	28.645800	2014	[157]
Rapana venosa	Black Sea and adjacent waters	43.921400	28.649700	2014	[157]
Rapana venosa	Black Sea and adjacent waters	43.902800	28.633600	2014	[157]
Rapana venosa	Black Sea and adjacent waters	43.894700	28.617800	2014	[157]
Rapana venosa	Black Sea and adjacent waters	44.014500	28.678100	2014	[157]
Rapana venosa	Black Sea and adjacent waters	44.005500	28.681000	2014	[157]
Rapana venosa	Black Sea and adjacent waters	43.982100	28.677600	2014	[157]
Rapana venosa	Black Sea and adjacent waters	43.943800	28.645800	2014	[157]
Rapana venosa	Black Sea and adjacent waters	43.931500	28.639000	2014	[157]
Rapana venosa	Black Sea and adjacent waters	44.653200	29.023400	2015	[157]
Rapana venosa	Black Sea and adjacent waters	45.989900	30.711100	2016	[157]
Rapana venosa	Black Sea and adjacent waters	41.558000	41.551900	2016	[157]
Rapana venosa	Black Sea and adjacent waters	42.376900	41.533700	2016	[157]
Rapana venosa	Mediterranean Sea	43.509700	13.652200	2017	[157]
Rapana venosa	Mediterranean Sea	38.466100	27.080300	2017	[157]
Rapana venosa	Black Sea and adjacent waters	44.208900	28.657400	2020	[157]
Rapana venosa	Black Sea and adjacent waters	44.204700	28.656000	2020	[157]
Rapana venosa	Black Sea and adjacent waters	44.205500	28.656900	2020	[157]
Rapana venosa	Black Sea and adjacent waters	44.644100	29.188500	2020	[157]
Rapana venosa	Black Sea and adjacent waters	44.649200	29.309500	2020	[157]
Rapana venosa	Black Sea and adjacent waters	44.994900	29.699200	2020	[157]
Rapana venosa	Black Sea and adjacent waters	44.582600	29.028600	2020	[157]
Rapana venosa	Mediterranean Sea	43.986100	12.697200	2021	[157]
Rapana venosa	Mediterranean Sea	43.548500	13.628300	2021	[157]
Rapana venosa	Mediterranean Sea	44.079619	12.575119	1985	[158]
Rapana venosa	Northwest Europe	42.636944	-8.978889	2007	[159]
Rapana venosa	Black Sea and adjacent waters	43.752180	28.578750	2018	[160]
Rapana venosa	Mediterranean Sea	41.094400	19.456100	2011	[161]

SPECIES	AREA	LATITUDE	LONGITUDE	YEAR	REFERENCE
Rapana venosa	Mediterranean Sea	43.966600	12.765600	1975	[162]
Rapana venosa	Black Sea and adjacent waters	45.101600	36.458900	2020	[163]
Rapana venosa	Black Sea and adjacent waters	45.077200	36.424200	2020	[163]
Rapana venosa	Black Sea and adjacent waters	45.058900	36.374500	2020	[163]
Rapana venosa	Black Sea and adjacent waters	45.032800	35.387500	2020	[163]
Rapana venosa	Black Sea and adjacent waters	44.958900	35.353200	2020	[163]
Rapana venosa	Black Sea and adjacent waters	44.924700	35.243800	2020	[163]
Rapana venosa	Black Sea and adjacent waters	45.306900	36.621700	1995	[164]
Rapana venosa	Black Sea and adjacent waters	43.588500	39.706900	1995	[164]
Rapana venosa	Black Sea and adjacent waters	45.115600	36.690000	2005	[164]
Rapana venosa	Black Sea and adjacent waters	45.068700	36.991200	2005	[164]
Rapana venosa	Black Sea and adjacent waters	45.001500	37.192200	2005	[164]
Rapana venosa	Black Sea and adjacent waters	44.883500	37.293900	2005	[164]
Rapana venosa	Black Sea and adjacent waters	44.753500	37.375300	2005	[164]
Rapana venosa	Black Sea and adjacent waters	44.660300	37.613500	2005	[164]
Rapana venosa	Black Sea and adjacent waters	44.629000	37.900500	2005	[164]
Rapana venosa	Black Sea and adjacent waters	44.454000	38.155400	2005	[164]
Rapana venosa	Black Sea and adjacent waters	44.349400	38.530300	2005	[164]
Rapana venosa	Black Sea and adjacent waters	44.306700	38.695800	2005	[164]
Rapana venosa	Black Sea and adjacent waters	44.249900	38.817300	2005	[164]
Rapana venosa	Black Sea and adjacent waters	44.087200	39.061500	2005	[164]
Rapana venosa	Black Sea and adjacent waters	43.893900	39.326300	2005	[164]
Rapana venosa	Black Sea and adjacent waters	43.746800	39.497100	2005	[164]
Rapana venosa	Black Sea and adjacent waters	43.588500	39.706900	2005	[164]
Rapana venosa	Black Sea and adjacent waters	43.473100	39.889600	2005	[164]
Rapana venosa	Black Sea and adjacent waters	43.473100	39.889600	2011	[164]
Rapana venosa	Black Sea and adjacent waters	40.926449	40.210968	2000	[165]
Rapana venosa	Black Sea and adjacent waters	40.926400	40.210900	1996	[166]
Rapana venosa	Black Sea and adjacent waters	41.017100	39.722300	2006	[167]
Rapana venosa	Black Sea and adjacent waters	40.988300	37.899300	2006	[167]

SPECIES	AREA	LATITUD E	LONGITUD E	YEA R	REFERENCE
Rapana venosa	Black Sea and adjacent waters	41.017100	39.722300	2006	[168]
Rapana venosa	Black Sea and adjacent waters	41.292100	36.361700	2006	[168]
Rapana venosa	Black Sea and adjacent waters	41.292100	36.361700	2011	[169]
Rapana venosa	Black Sea and adjacent waters	41.044400	40.518600	2003	[170]
Rapana venosa	Northwest Europe	46.145024	-1.184730	2021	[171]
Rapana venosa	Northwest Europe	43.668794	-1.429577	2022	[171]
Rapana venosa	Northwest Europe	44.671697	-1.117593	2022	[171]
Rapana venosa	Mediterranean Sea	44.265000	12.443333	2002	[172]
Rapana venosa	Mediterranean Sea	44.207600	12.407800	2002	[172]
Rapana venosa	Mediterranean Sea	44.223333	12.381667	2001	[173]
Rapana venosa	Mediterranean Sea	44.223333	12.443333	2001	[173]
Rapana venosa	Mediterranean Sea	44.265000	12.381667	2001	[173]
Rapana venosa	Mediterranean Sea	44.265000	12.443333	2001	[173]
Rapana venosa	Mediterranean Sea	44.265000	12.443333	2001	[173]
Rapana venosa	Mediterranean Sea	44.203333	12.411667	2001	[173]
Rapana venosa	Mediterranean Sea	45.701825	13.178374	2004	[174]
Rapana venosa	Mediterranean Sea	45.251425	12.312941	2004	[174]
Rapana venosa	Mediterranean Sea	44.738144	12.386595	2004	[174]
Rapana venosa	Mediterranean Sea	44.203333	12.411667	2004	[174]
Rapana venosa	Mediterranean Sea	43.852905	13.026170	2004	[174]
Rapana venosa	Black Sea and adjacent waters	45.329700	36.655500	2014	[175]
Rapana venosa	Black Sea and adjacent waters	45.246000	36.595400	2014	[175]
Rapana venosa	Black Sea and adjacent waters	45.451030	36.856490	2014	[175]
Rapana venosa	Black Sea and adjacent waters	45.140280	36.633610	2014	[175]
Rapana venosa	Black Sea and adjacent waters	45.101600	36.458900	2014	[175]
Rapana venosa	Black Sea and adjacent waters	45.423611	36.576389	2014	[175]
Rapana venosa	Black Sea and adjacent waters	45.244564	36.501356	2014	[175]
Rapana venosa	Black Sea and adjacent waters	45.306900	36.621700	2015	[175]
Rapana venosa	Black Sea and adjacent waters	45.417900	36.753900	2015	[175]
Rapana venosa	Black Sea and adjacent waters	45.237500	36.607200	2015	[175]

SPECIES	AREA	LATITUDE	LONGITUDE	YEAR	REFERENCE
Rapana venosa	Black Sea and adjacent waters	45.221500	36.609500	2015	[175]
Rapana venosa	Black Sea and adjacent waters	45.194000	36.591900	2015	[175]
Rapana venosa	Black Sea and adjacent waters	45.137400	36.628700	2015	[175]
Rapana venosa	Black Sea and adjacent waters	45.113500	36.443900	2015	[175]
Rapana venosa	Black Sea and adjacent waters	45.345500	36.601200	2015	[175]
Rapana venosa	Black Sea and adjacent waters	45.195280	36.598890	2015	[175]
Rapana venosa	Black Sea and adjacent waters	45.166000	36.417600	2015	[175]
Rapana venosa	Black Sea and adjacent waters	45.236300	36.422600	2015	[175]
Rapana venosa	Black Sea and adjacent waters	45.273300	36.439300	2015	[175]
Rapana venosa	Black Sea and adjacent waters	45.319400	36.492800	2015	[175]
Rapana venosa	Black Sea and adjacent waters	45.349700	36.530400	2015	[175]
Rapana venosa	Black Sea and adjacent waters	44.345070	28.692010	2017	[176]
Rapana venosa	Black Sea and adjacent waters	43.772300	28.579220	2017	[176]
Rapana venosa	Black Sea and adjacent waters	44.428690	28.771790	2013	[177]
Rapana venosa	Black Sea and adjacent waters	44.338900	28.701930	2013	[177]
Rapana venosa	Black Sea and adjacent waters	43.785540	28.586200	2013	[177]
Rapana venosa	Mediterranean Sea	43.914000	12.922100	1999	[178]
Rapana venosa	Black Sea and adjacent waters	41.723200	36.000600	2018	[179]
Rapana venosa	Black Sea and adjacent waters	41.694800	36.056500	2018	[179]
Rapana venosa	Black Sea and adjacent waters	41.476100	36.136200	2018	[179]
Rapana venosa	Black Sea and adjacent waters	41.430500	36.186400	2018	[179]
Rapana venosa	Black Sea and adjacent waters	41.017100	39.722300	1999	[180]
Rapana venosa	Black Sea and adjacent waters	45.027100	36.225500	2002	[181]
Rapana venosa	Black Sea and adjacent waters	44.928000	35.247800	1976	[182]
Rapana venosa	Black Sea and adjacent waters	44.611300	33.499300	2018	[183]
Rapana venosa	Black Sea and adjacent waters	42.817188	27.887134	1989	[184]
Rapana venosa	Black Sea and adjacent waters	43.588500	39.706900	1989	[184]
Rapana venosa	Black Sea and adjacent waters	45.254033	30.195656	2005	[185]
Rapana venosa	Black Sea and adjacent waters	45.259633	30.199031	2005	[185]
Rapana venosa	Black Sea and adjacent waters	45.259982	30.205000	2005	[185]

SPECIES	AREA	LATITUDE	LONGITUDE	YEAR	REFERENCE
Rapana venosa	Black Sea and adjacent waters	45.258446	30.210400	2005	[185]
Rapana venosa	Black Sea and adjacent waters	45.254302	30.209267	2005	[185]
Rapana venosa	Black Sea and adjacent waters	45.248465	30.203890	2005	[185]
Rapana venosa	Mediterranean Sea	43.474996	5.005145	2020	[186]
Rapana venosa	Black Sea and adjacent waters	45.252278	30.202722	2022	[187]
Rapana venosa	Mediterranean Sea	45.516567	13.458583	2004	[188]
Rapana venosa	Black Sea and adjacent waters	43.000000	40.533333	1949	[189]
Rapana venosa	Black Sea and adjacent waters	43.000000	40.533333	1950	[189]
Rapana venosa	Black Sea and adjacent waters	44.638500	29.160300	1970	[190]
Rapana venosa	Black Sea and adjacent waters	44.359900	28.712800	1970	[190]
Rapana venosa	Black Sea and adjacent waters	44.338900	28.701900	1997	[190]
Rapana venosa	Black Sea and adjacent waters	44.263000	28.628600	1997	[190]
Rapana venosa	Black Sea and adjacent waters	44.186700	28.662800	1997	[190]
Rapana venosa	Black Sea and adjacent waters	44.071800	28.645400	1997	[190]
Rapana venosa	Black Sea and adjacent waters	44.030900	28.666500	1997	[190]
Rapana venosa	Black Sea and adjacent waters	43.955800	28.643900	1997	[190]
Rapana venosa	Black Sea and adjacent waters	43.813500	28.592400	1997	[190]
Rapana venosa	Black Sea and adjacent waters	43.752100	28.578700	1997	[190]
Rapana venosa	Black Sea and adjacent waters	44.428600	28.771700	2005	[190]
Rapana venosa	Black Sea and adjacent waters	44.684100	29.002300	2008	[190]
Rapana venosa	Black Sea and adjacent waters	44.397600	28.738100	2008	[190]
Rapana venosa	Mediterranean Sea	42.809289	10.140072	1978	[191]
Rapana venosa	Black Sea and adjacent waters	46.441300	30.772600	2013	[192]
Rapana venosa	Black Sea and adjacent waters	43.177433	27.929150	2015	[193]
Rapana venosa	Black Sea and adjacent waters	43.176933	27.930367	2015	[193]
Rapana venosa	Black Sea and adjacent waters	43.177417	27.932400	2015	[193]
Rapana venosa	Black Sea and adjacent waters	43.176300	27.932150	2016	[193]
Rapana venosa	Black Sea and adjacent waters	43.176517	27.932050	2016	[193]
Rapana venosa	Black Sea and adjacent waters	43.176917	27.931667	2016	[193]
Rapana venosa	Black Sea and adjacent waters	43.103500	27.921667	2016	[194]

SPECIES	AREA	LATITUDE	LONGITUDE	YEAR	REFERENCE
Rapana venosa	Black Sea and adjacent waters	43.103333	27.937833	2016	[194]
Rapana venosa	Black Sea and adjacent waters	43.103167	27.951833	2016	[194]
Rapana venosa	Northwest Europe	42.583647	-8.820472	2005	[195]
Rapana venosa	Black Sea and adjacent waters	43.033500	27.889800	1964	[196]
Rapana venosa	Black Sea and adjacent waters	42.880400	27.901600	1964	[196]
Rapana venosa	Black Sea and adjacent waters	42.698200	27.899900	1964	[196]
Rapana venosa	Black Sea and adjacent waters	42.658600	27.750400	1964	[196]
Rapana venosa	Black Sea and adjacent waters	42.562100	27.615600	1964	[196]
Rapana venosa	Black Sea and adjacent waters	42.506400	27.486000	1964	[196]
Rapana venosa	Black Sea and adjacent waters	42.468500	27.553300	1964	[196]
Rapana venosa	Black Sea and adjacent waters	43.189200	27.977900	1964	[196]
Rapana venosa	Black Sea and adjacent waters	40.925931	40.203175	2020	[197]
Rapana venosa	Black Sea and adjacent waters	41.633056	35.532222	2013	[198]
Rapana venosa	Black Sea and adjacent waters	41.885800	28.031600	2021	[199]
Rapana venosa	Mediterranean Sea	43.916900	12.919100	1989	[200]
Rapana venosa	Mediterranean Sea	36.313800	33.887700	1989	[200]
Rapana venosa	Black Sea and adjacent waters	41.183085	29.076601	1990	[200]
Rapana venosa	Black Sea and adjacent waters	41.235400	29.109400	1990	[200]
Rapana venosa	Black Sea and adjacent waters	40.972100	27.513400	1990	[200]
Rapana venosa	Mediterranean Sea	44.079600	12.575100	1997	[200]
Rapana venosa	Black Sea and adjacent waters	40.972100	27.513400	2012	[200]
Rapana venosa	Black Sea and adjacent waters	40.959900	27.933700	2012	[200]
Rapana venosa	Black Sea and adjacent waters	41.259300	36.494500	2014	[201]
Rapana venosa	Black Sea and adjacent waters	41.341800	36.287700	2014	[201]
Rapana venosa	Black Sea and adjacent waters	43.000000	40.533333	1990	[202]
Rapana venosa	Black Sea and adjacent waters	43.000000	40.533333	1990	[202]
Rapana venosa	Black Sea and adjacent waters	43.000000	40.533333	1990	[202]
Rapana venosa	Black Sea and adjacent waters	44.883500	37.293900	1988	[203]
Rapana venosa	Black Sea and adjacent waters	44.557900	38.022800	1988	[203]
Rapana venosa	Black Sea and adjacent waters	41.662600	41.632300	1988	[203]

SPECIES	AREA	LATITUDE	LONGITUDE	YEAR	REFERENCE
Rapana venosa	Black Sea and adjacent waters	45.194000	36.591900	1990	[203]
Rapana venosa	Black Sea and adjacent waters	44.995700	35.839100	1994	[203]
Rapana venosa	Black Sea and adjacent waters	45.101600	36.458900	1994	[203]
Rapana venosa	Mediterranean Sea	44.845398	12.435761	2024	Luca Castriota. unpublished observation
